# Dehydration triggers ecdysone-mediated recognition-protein priming and elevated anti-bacterial immune responses in *Drosophila* Malpighian tubule renal cells

**DOI:** 10.1186/s12915-018-0532-5

**Published:** 2018-05-31

**Authors:** Wenjing Zheng, Florentina Rus, Ana Hernandez, Ping Kang, William Goldman, Neal Silverman, Marc Tatar

**Affiliations:** 1 Division of Biology and Medicine, Brown University, Providence, RI USA; 2 Department of Medicine, Division of Infectious Diseases, University of Massachusetts, Medical School, Worcester, MA USA; 3 Department of Microbiology and Immunology, University of North Carolina, Chapel Hill, NC USA

**Keywords:** Drosophila Malpighian tubules, desiccation, ecdysone, innate immune aging, immunosenescence

## Abstract

**Background:**

*Drosophila* is a powerful model for the study of factors modulating innate immunity. This study examines the effect of water-loss dehydration on innate immune responsiveness in the *Drosophila* renal system (Malpighian tubules; MTs), and how this leads to elevated host defense and contributes to immunosenescence.

**Results:**

A short period of desiccation-elevated peptidoglycan recognition protein-LC (*PGRP-LC*) expression in MTs, increased antimicrobial peptide (*AMP*) gene induction, and protected animals from bacterial infection. We show that desiccation increased ecdysone synthesis in MTs, while inhibition of ecdysone synthesis or ecdysone receptor expression, specifically within MTs, prevented induction of PGRP-LC and reduced protection from bacterial infection. Additionally, aged flies are constitutively water-stressed and have elevated levels of ecdysone and PGRP-LC. Conversely, adults aged at high relative humidity show less water loss and have reduced expression of *PGRP-LC* and *AMPs*.

**Conclusions:**

The *Drosophila* renal system is an important contributor to host defense and can modulate immune responses in an organ autonomous manner, responding to environmental changes such as desiccation. Desiccation primes immune responsiveness by elevating *PGRP-LC* expression specifically in MTs. In response to desiccation, ecdysone is produced in MTs and acts in a paracrine fashion to increase *PGRP-LC* expression, immune responsiveness, and improve host defense. This activity of the renal system may contribute to the immunosenescence observed in Drosophila.

**Electronic supplementary material:**

The online version of this article (10.1186/s12915-018-0532-5) contains supplementary material, which is available to authorized users.

## Background

In *Drosophila*, the humoral immune response is characterized by the rapid induction of a battery of antimicrobial peptides (AMPs). These endogenous antimicrobials are expressed at nearly undetectable levels in the blood (or hemolymph) of healthy, young adult flies but are transcriptionally induced to massive levels following systemic microbial infection. This response is triggered by microbial cell walls, peptidoglycans from bacteria or beta-glucans from fungi, and is regulated by two nuclear factor kappa-light-chain-enhancer of activated B cells (NF-κB) signaling pathways, the Toll and Imd pathways [1, 2].

The Imd pathway responds to diaminopimelic acid-type peptidoglycan (DAP-type PGN) from the cell wall of Gram-negative and certain types of Gram-positive bacteria. DAP-type PGN is sensed by two receptors, both members of the peptidoglycan recognition protein (PGRPs) family of receptors. PGRP-LC is a transmembrane receptor found on the cell surface, while PGRP-LE is a cytosolic receptor [3]. DAP-type PGN ligation of either of these receptors is sufficient to activate Imd signaling and massive *AMP* gene induction. In particular, these receptors trigger the formation of a functional amyloidal signaling platform, involving the factor Imd [4], which in turns leads to K63-ubiquitination, activation of the *Drosophila* kinases TGF-β activated kinase 1 and I Kappa B Kinase, and ultimately the cleavage and nuclear translocation of the NF-κB precursor Relish [5–7]. Relish directly induces *AMP* gene expression.

Developmental and environmental signals modulate the activity and sensitivity of the Imd immune response. In particular, the steroid hormone 20-hydroxyecdysone (20E) controls the expression of the key PGN-sensing receptor PGRP-LC [8]. In earlier work, we established that 20E, signaling through a canonical nuclear hormone receptor pathway, is required for *PGRP-LC* expression both in cultured cells and in adult flies. Given the central role PGRP-LC plays in sensing systemic bacterial infections, steroid hormone regulation provides a significant degree of modulation on the ability of cells or animals to respond to bacterial infection. It is clear from earlier literature that this regulatory network provides developmental modulation to Imd responses. In particular, early third instar larvae are largely unresponsive to challenge with DAP-type PGN-containing microbes, while wandering larvae, which have received a large bolus of 20E as part of their developmental program, are highly responsive to these infections [9].

In adult flies, it has been suggested that 20E also serves as a stress-responsive steroid hormone, analogous to adrenal-produced, stress-responsive hormones (such as glucocorticoids) in mammals [10–13]. In this study, we have examined how 20E modulates the immune response following the stress caused by a brief period of desiccation. Given the physiological role for the Malpighian tubules (MTs, the insect renal system) in solute homeostasis [14], this immune responsive organ was the focus of investigation. We found that MT autonomously exhibited dehydration-induced steroid-mediated priming of the Imd pathway. This priming response required 20E-mediated upregulation of the innate immune recognition receptor *PGRP-LC*, thus termed recognition-protein priming. Aged flies are known to naturally become dehydrated [15, 16], and experimental rehydration ameliorated the elevated Imd activity typically observed in older animals, suggesting a link between dehydration-induced recognition-protein priming and immunosenescence. Moreover, dehydration stress-induced steroid-mediated recognition-protein priming, specifically in the MTs, provided enhanced protection to systemic bacterial infection in adult animals. These data demonstrate the important role of steroid-mediated recognition-protein priming in modulating the immune response of adult *Drosophila*, shaping host defense based on life history and environmental conditions.

## Results

### Desiccation increases immune sensitivity in the *Drosophila* renal system

**Fig. 1:**
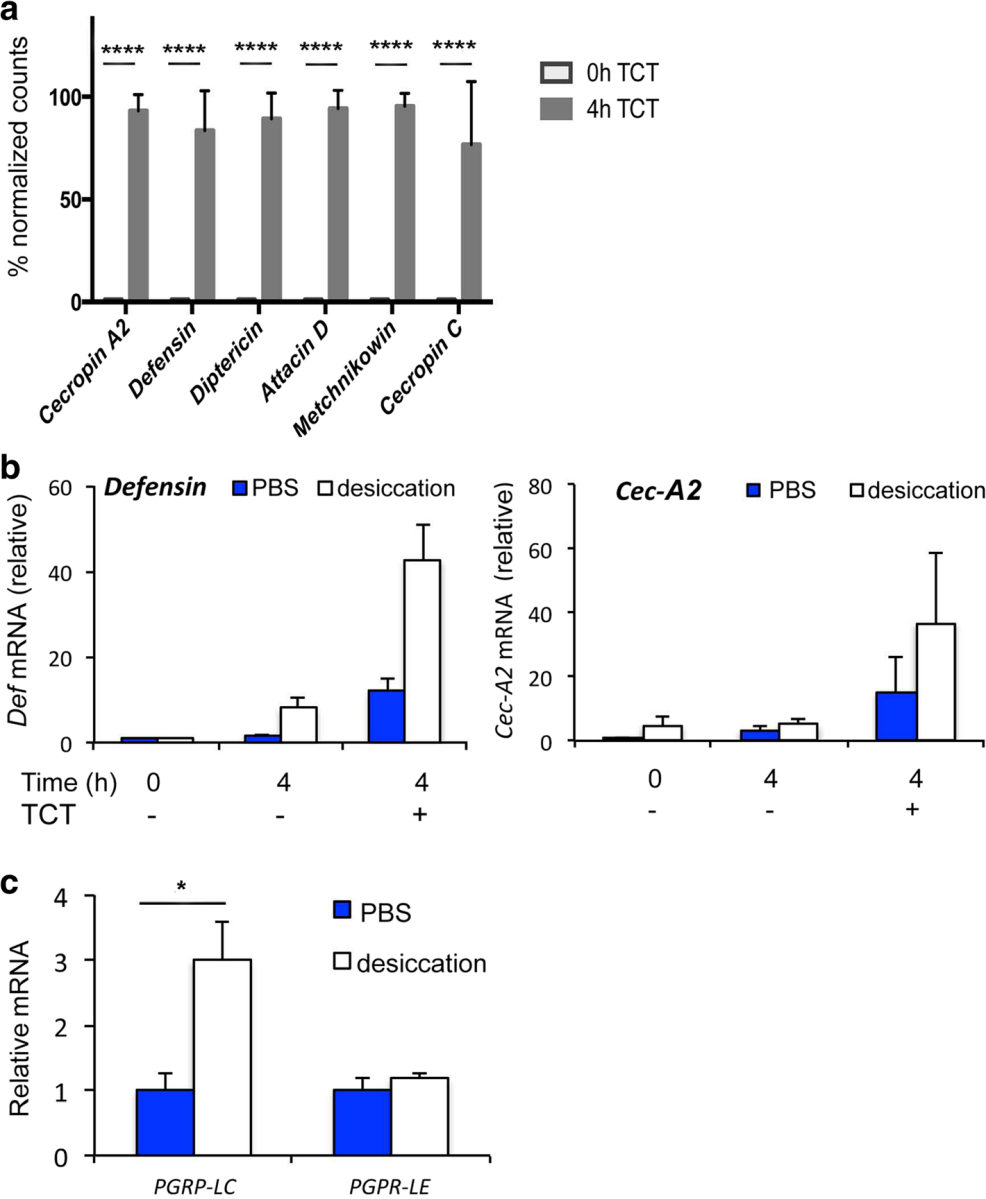
TCT and desiccation stress induce multiple AMP genes in Malpighian tubules (MTs). **a** Nanostring nCounter analysis of AMP transcripts from isolated MTs of *wDah* females (7 days old) exposed to TCT for 4 h compared to unstimulated tubules. The mean of four independent biological replicates, harvested on separate days, is shown, and error bars represent standard error of the mean. Statistical significance was calculated by two-way ANOVA and Sidak’s multiple comparison test; **p < 0.01. **b** mRNA expression (means with SEM) for *Defensin* and *Cecropin-A2* (*Cec-A2*) measured by qRT-PCR relative to control (time 0, no TCT). TCT induces *Defensin* (Likelihood ratio test, DF = 1, χ^2^ = 9.32, *p* = 0.0023) and *Cec-A2* (Likelihood ratio test, DF = 1, χ^2^ = 6.15, *p* = 0.013). Generalized Linear Model estimated by maximum likelihood with TCT and desiccation as main effects and TCT x desiccation as interaction effect, *N* = 12; desiccation and TCT significantly interact to amplify *Defensin* mRNA (TCT x desiccation χ^2^ = 3.96 *p* = 0.046). The desiccation-induced increase for *Cec-A2* did not reach significance (TCT x desiccation χ^2^ = 1.03, *p* = 0.31). **c** qRT-PCR analysis of *PGRP-LC* and *PGRP-LE* expression from MTs, normalized to PBS-treated control. *p < 0.05; means with SEM, *N* = 6; *t* test; PGRP-LC *p* = 0.038, PGRP-LE *p* = 0.27

It was previously shown that *Drosophila* MTs are autonomously immune responsive with robust *AMP* gene induction following immune challenge in intact animals and in dissected tubules [3, 17, 18]*.* In MTs and other immune responsive tissues, tracheal cytotoxin (TCT), a monomeric fragment of DAP-type PGN, activates Imd signaling [3, 19]. Thus, to characterize the innate immune response in MTs, we challenged MTs excised from wild-type (*wDah*) females with TCT and measured *AMP* gene expression using NanoString nCounter. As shown here, TCT induced robust expression of multiple *AMP* genes in MTs dissected from 7-day-old females (Fig. 1a, Additional file 1: Figure S1).

In preliminary studies with this ex vivo MT assay, we noticed a high degree of variability in the amplitude of AMP induction. This led us to hypothesize that animals may be exposed to relatively brief periods of dehydration during handling on the CO_2_ anesthesia ‘fly pad’, which could be affecting immune response amplitudes. An experimental protocol was designed to directly test this hypothesis. Without anesthesia, 7-day-old females were transferred to vials and maintained in PBS (isotonic to fly hemolymph) or in a dry vial (desiccation) for 2 h (both conditions without food). RNA was isolated from freshly dissected MTs (time 0), and from dissected MTs incubated for 4 h with or without TCT. *Defensin* and *Cecropin-A2* (*Cec-A2*) mRNAs were quantified. MTs stimulated with TCT strongly induced *Defensin* and *Cec-A2* compared to freshly dissected MTs or to mock-treated MTs (Fig. 1b). For *Defensin*, desiccation triggered a modest increase in expression in the mock-treated samples and amplified the induction upon TCT treatment (two-way ANOVA, χ^2^_(desiccation x TCT)_ = 3.96, *p* < 0.05). For *Cec-A2*, the effect of desiccation trended in the same direction, but did not reach statistical significance (χ^2^_(desiccation x TCT)_ = 1.31, *p* = 0.31). In a second similarly designed experiment, RNA was extracted from four biologically independent sets of MT, isolated from control (PBS) or desiccated *wDah* animals, and analyzed by Nanostring nCounter for the expression of multiple AMPs and other immune related genes (Heat Map in Additional file 2: Figures S2). In this analysis, 13 AMP genes were significantly upregulated either by desiccation alone or upon TCT-challenge following desiccation. Many of these AMP genes showed significant interactions between desiccation and TCT (side table in Additional file 3: Figure S3, after Bonferroni correction for multiple comparisons). Together, these data demonstrate that desiccation robustly induces *AMP* gene expression within the MT, and frequently increases the sensitivity to TCT.

### Desiccation triggers increased expression of the innate immune recognition protein via steroid hormone signaling

In MTs, TCT is a direct agonist of both the cell surface receptor PGRP-LC and the cytosolic receptor PGRP-LE [19–23]. Desiccation caused a significant increase in *PGRP-LC* mRNA expressed in MT, while *PGRP-LE* expression was unchanged (Fig. 1c). Overall, MT from adults held without water for a short duration increased expression of *PGRP-LC* and their capacity to induce *AMP* gene expression.

**Fig. 2:**
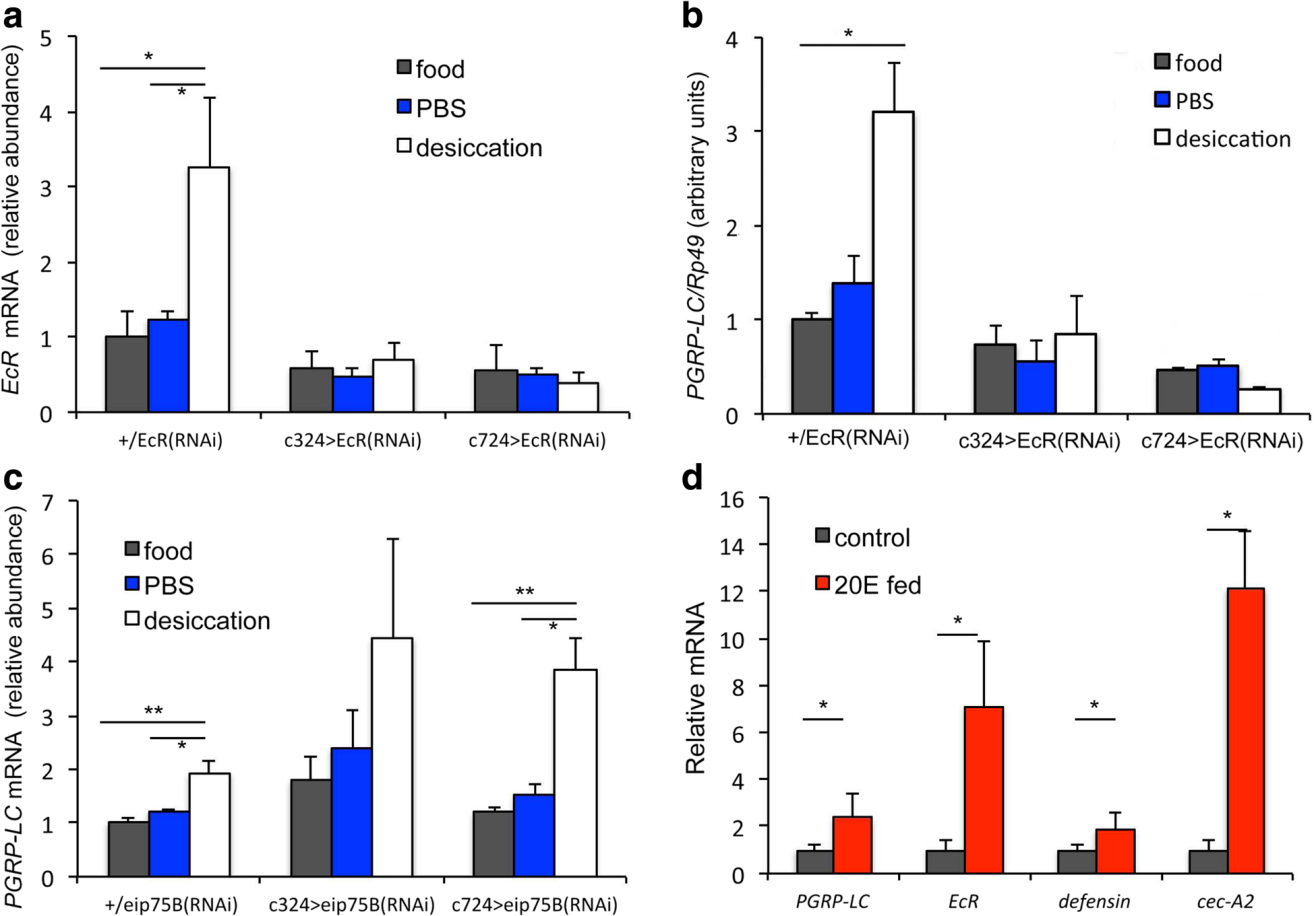
Malpighian tubule (MT) *EcR* and *PGRP-LC* induced by desiccation and 20E feeding. Relative mRNA of (**a**) *EcR* and (**b**) *PGRP-LC* in control genotype (+/*EcR*(RNAi)) and with *EcR*(RNAi) expressed in stellate cells (c724>) or principal cells (c324>). **c** Relative mRNA of *PGRP-LC* in control genotype (+/*eip75B*(RNAi)) and when *eip75B*(RNAi) is expressed in stellate cells (c724>) or principal cells (c324>). **a–c** Mean with SEM of three independent biological samples for each genotype, normalized relative to food treatment, wild-type genotype. Within each genotype: one-way ANOVA with treatment as main effect (*N* = 9), *p < 0.05, **p < 0.01 and Tukey’s post hoc comparison. **d** Relative mRNA for *PGRP-LC*, *EcR*, *Defensin*, and *Cec-A2* from MTs of adults fed 20E, each normalized to corresponding control. Mean of three independent biological samples (SEM); *t* test for each gene with Holm–Bonferroni Sequential correction *p < 0.05

Among its many functions [24], ecdysone modulates AMP expression in larvae, cultured cells, pupae, and adults [8, 9, 25–28]. In particular, 20E regulates *PGRP-LC* expression [8]. Ecdysone also induces expression and synthesis of its nuclear hormone receptor, ecdysone receptor (EcR) [29]. Here, we found elevated *EcR* mRNA levels in MTs of desiccated flies, compared to PBS-treated or conventionally reared animals (Fig. 2a). In addition, ecdysone is known to activate an EcR-responsive reporter in adult tissues including the MT [30]. Therefore, we tested if *EcR* is required for desiccation to induce *PGRP-LC* in MTs*. EcR* RNAi expressed in stellate cells (*c724*-Gal4) or principal cells (*c324*-Gal4) blocked the ability of desiccation to induce *EcR* or *PGRP-LC* expression in MTs (Fig. 2a, b). In a complementary fashion, *ecdysone induced protein 75* (*Eip75*), a negative regulator of ecdysone-mediated signaling, was targeted via tissue-specific RNAi. Knockdown in stellate cells (via *c724*-Gal4/UAS-*eip75B*(RNAi)) significantly amplified the ability of desiccation to increase *PGRP-LC* mRNA relative to control genotype (*β*_genotype x desiccation_ = −0.018, χ^2^ = −3.50, *p* < 0.01), while *c324*-Gal4/UAS-*eip75B*(RNAi) trended in the same direction, but did not reach statistical significance (Fig. 2c).

Ecdysone has a myriad of activities beyond its classic role in controlling the insect life cycle. For example, 20E drives *PGRP-LC* expression in S2* cells [8]. Additionally, 20E fed to adult *Drosophila* restores normal aging to otherwise long-lived *EcR* mutant females, restores courtship memory defects and promotes sleep [12, 31, 32]. Here, 20E fed to females induced the expression of *EcR* and *PGRP-LC* mRNA within MTs, as well as the expression of *Defensin* and *Cec-A2* even without external immune challenge (Fig. 2d).

**Fig. 3:**
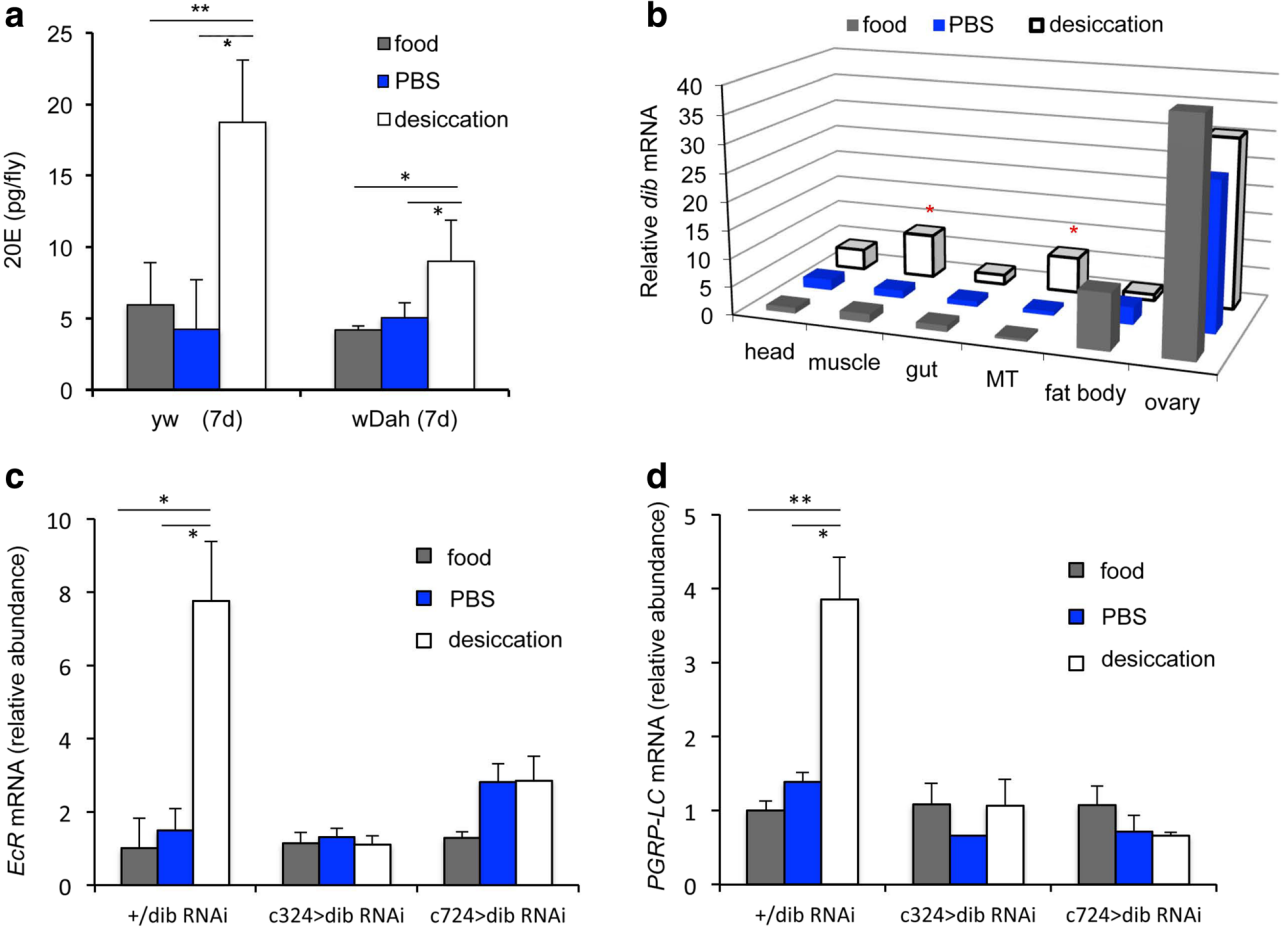
Water-loss dehydration increases ecdysone and Malpighian tubule (MT) expression of ecdysone biosynthetic genes in young females (7 days). **a** 20E titer (pg/fly) (mean and SEM among three biological replicates, each of 25–30 pooled females) from whole females of two genetic backgrounds (*yw* and *wDah*) with control (food or PBS) and desiccation treatments. Independent biological replicates were prepared on separate days. One-way ANOVA within each genotype, Tukey’s post hoc comparison: *p < 0.05, **p < 0.01. **b** Relative *disembodied* (*dib*) mRNA in female tissues from control (food or PBS) and desiccation treatments. All values normalized to mean value from food treatment, head sample; mean values of three biological replicates, each containing the specific tissue from 15 females. Statistical comparisons are made within each tissue by *t* test for difference between desiccation and PBS treatment: dib mRNA was significantly elevated in muscle and MT (*p < 0.05 with Holm–Bonferroni Sequential correction). **c** Relative mRNA for *EcR* and (**d**) *PGRP-LC* in control genotype (+/*dib*(RNAi)) and when *dib* is depleted in stellate cells (c724>) or principal cells (c324>). Values are mean and SEM of three independent biological replicates, normalized relative to food treatment, wild-type for each mRNA type. For each genotype: one-way ANOVA with Tukey’s post hoc comparison. For the control genotype (+/dib RNAi) a significant difference was observed between the flies exposed to desiccation compared to those exposed to normal food or PBS (*p < 0.05, **p < 0.01). However, no significant differences were observed when *EcR* or *PGRP-LC* were knocked down (*c324* > RNAi and *c724* > RNAi); *EcR*-RNAi *F* = 0.52, *p* = 0.62 and *F* = 4.43, *p* = 0.066; *PGRP-LC*-RNAi *F* = 0.39, *p* = 0.70 and *F* = 0.54, *p* = 0.61

Ecdysone signaling appears to drive the expression of *PGRP-LC* during desiccation stress, and thus prime tubules to express higher levels of AMPs (‘recognition-protein priming’). These results suggest that desiccation stress should increase 20E levels. Measured from whole animals of two genetic backgrounds (*yw* and *wDah*), 20E was elevated after 2 h of desiccation (Fig. 3a). To determine where this 20E is produced, the expression of ecdysone biosynthetic enzyme genes (Halloween cytochrome P450s) was quantified from different tissues (Fig. 3b and Additional file 4: Figure S4, all expression was normalized relative to the head sample in the food control group). Desiccation upregulated Halloween genes in several somatic tissues. The most striking effects (Fig. 3b) were observed for *disembodied* (*dib*), a cytochrome P450 with ecdysteroid 22-hydroxylase activity; control-treated females expressed *dib* strongly in ovaries (via follicle cells [33]) and modestly in fat body, while little *dib* mRNA expression was observed in other tissues. However, upon desiccation, *dib* mRNA was significantly increased in muscle and MTs. Accordingly, when *dib* mRNA was reduced by RNAi in stellate or principal cells (Additional file 5: Figure S5), *EcR* and *PGRP-LC* expression was not induced in MTs by desiccation relative to food or PBS controls (Fig. 3c, d).

Together, these data show that desiccation controls ecdysone synthesis within MTs, which locally regulates recognition-protein priming. On the other hand, females maintained for 2 h without food (PBS controls) produced significantly less *dib* mRNA in the fat body relative to food controls (*p* = 0.019). This effect on fat body expression of *dib* appears to be due to a starvation response rather than a desiccation response. No significant differences in *dib* expression in ovaries were observed among treatments.

### Aged flies exhibit desiccation-induced ecdysone-mediated recognition-protein priming

**Fig. 4:**
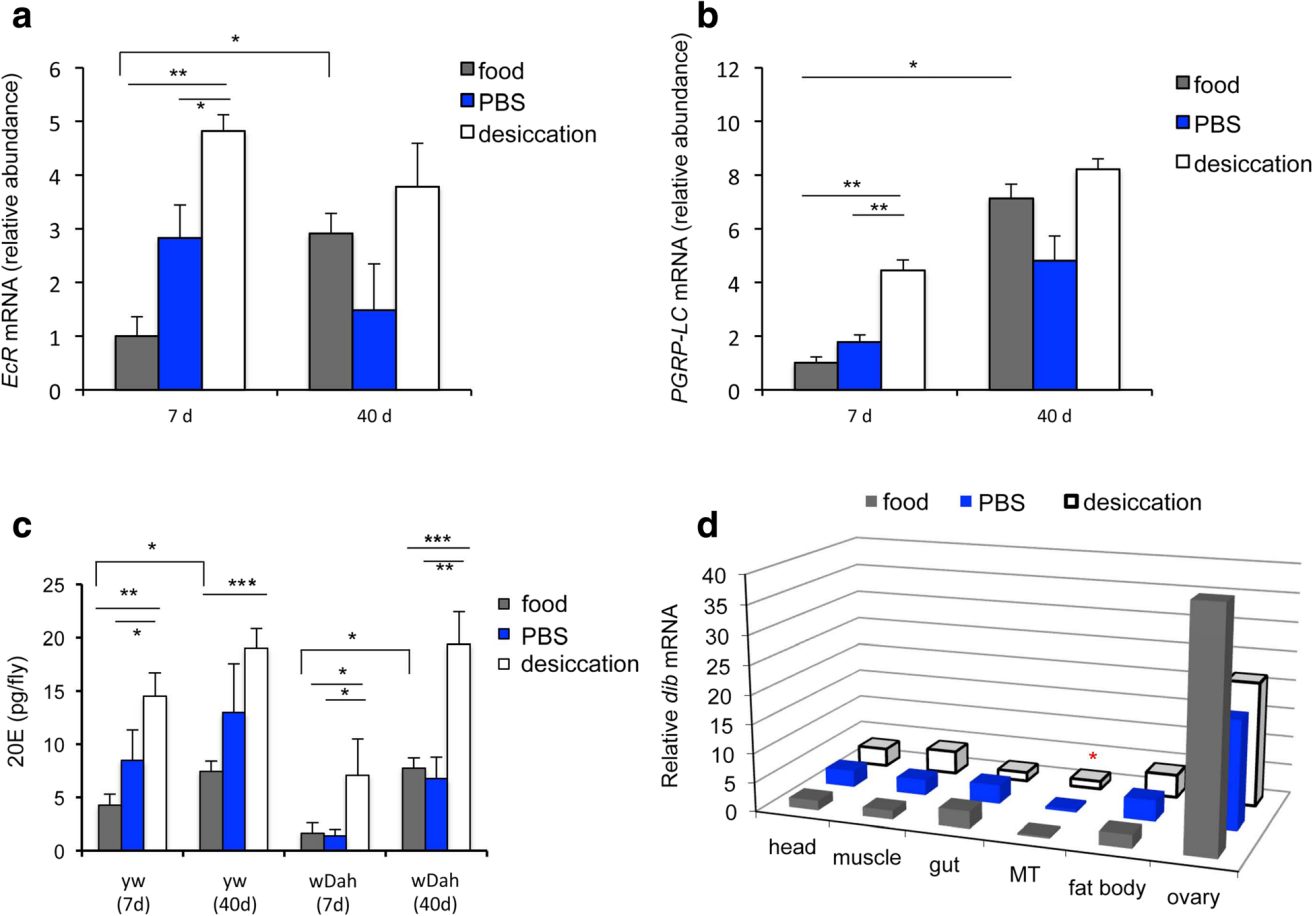
Peptidoglycan recognition-protein priming increases with age. (**a**) *EcR* and (**b**) *PGRP-LC* mRNA from Malpighian tubules (MTs) of young (7 days) and old (40 days) *yw* females in control (food, PBS) and desiccation treatments. Expression was normalized relative to young flies in the food treatment control; mean of three independent biological samples with SEM; two-way ANOVA with age and treatment as main effects, Tukey’s post hoc comparison, *p < 0.05, **p < 0.01. **c** 20E extracted from 7- and 40-day-old wild-type females (*yw* and *wDah*) in control (food, PBS) and desiccation treatments; mean of three independent biological samples with SEM. Within each genotype, two-way ANOVA with age and treatment as main effects, Tukey’s post hoc comparison, *p < 0.05, **p < 0.01, ***p < 0.001. **d** *Disembodied* (*dib*) mRNA from specific tissues of old females (40 days) in control (food, PBS) and desiccation treatments; normalized relative to food treatment, head tissue of young females; mean of three independent biological samples for each tissue type. Statistical comparisons within each tissue by *t* test for difference between desiccation and PBS treatment: dib mRNA was significantly elevated in MTs (*p < 0.05 with Holm-Bonferroni Sequential correction)

Interestingly, water loss rates and *AMP* gene expression both increase in aged *Drosophila* [16, 34–36]. In addition to higher AMP expression at baseline, older flies also induce higher levels of *AMP* gene expression when challenged with live bacteria [28, 35], suggesting that recognition receptor expression could be affected by age. Indeed, expression of *PGRP-LC* as well as *EcR* was increased in MT from flies at age 40 days (Fig. 4a, b). Experimental dehydration did not further elevate *PGRP-LC* and *EcR* mRNAs in aged animals, in contrast to their induction in young flies. Ecdysone titers measured from aged flies were also significantly elevated relative to young females in control (food) conditions, while desiccation increased 20E in both young and old animals (Fig. 4c). These data suggest that aging is associated with recognition-protein priming mediated through elevated ecdysone.

To identify the source of ecdysone in aged flies, tissue was dissected from 40 day females exposed to control (food, PBS) and dehydration treatments. *Dib* mRNA was quantified relative to young females. Relative to young females, old females expressed *dib* in similar but muted patterns (Fig. 4d, compared to Fig. 3b). *dib* was still expressed in ovaries of old, food-reared females, although in old females, fat body showed lower expression under all conditions. Old females under control conditions (PBS) expressed somewhat more *dib* than young females in head (1.83 fold), muscle (1.71 fold), and gut (2.97 fold) (each tissue *p* < 0.03). Expression of *dib* was similarly low in the MT of young and old control females, but significantly induced by desiccation in old MTs (3.5 fold), as observed in young females, and consistent with the ecdysone titers.

**Fig. 5:**
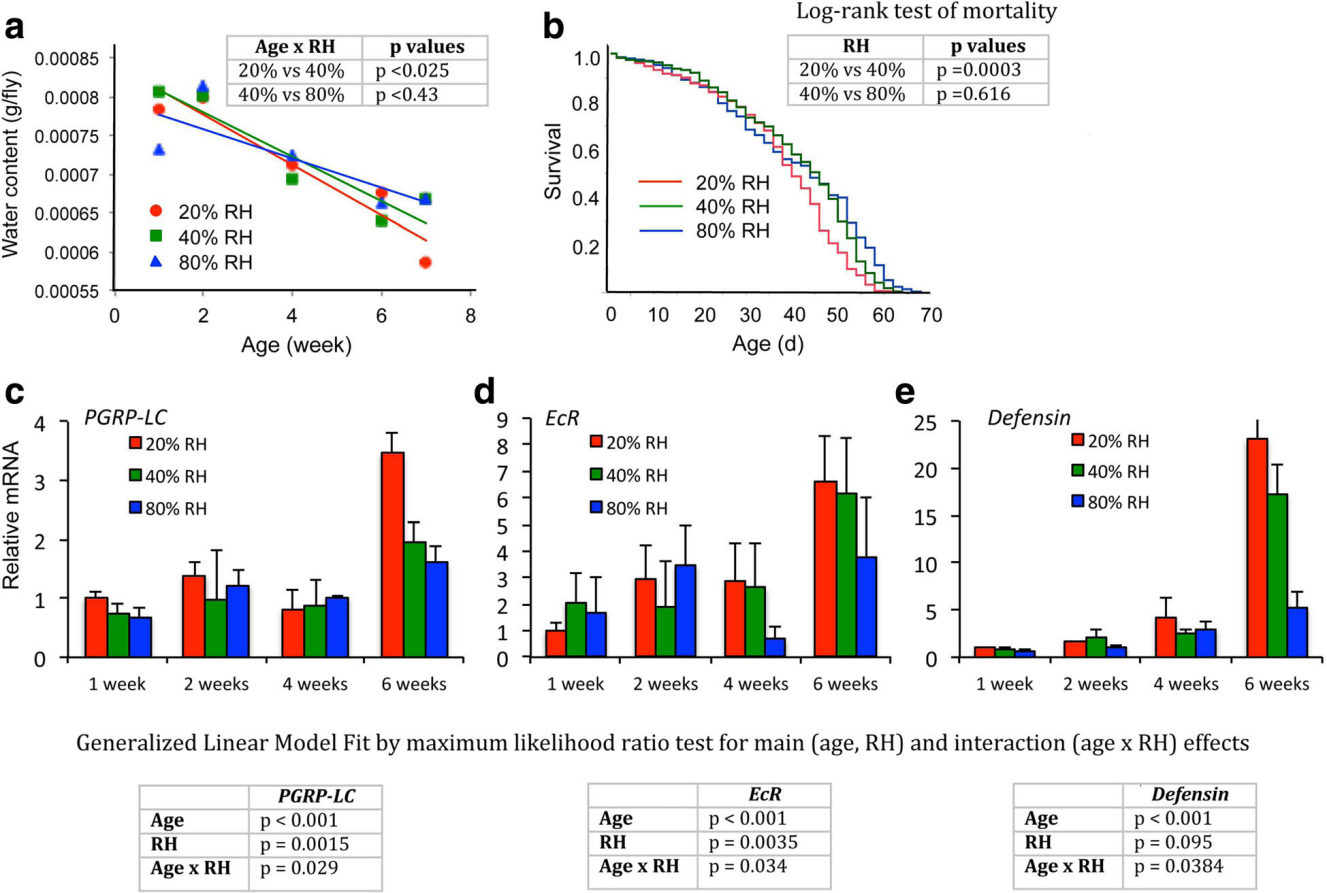
High relative humidity (RH) reduces water loss with age and slows innate immune aging. **a** Water content per adult female, cohorts sampled from age 1 to 7 weeks old; cohorts maintained at 20%, 40%, and 80% RH. Trajectory of water content among RH groups analyzed with Generalized Linear Model Fit by maximum likelihood ratio test. For main effects: age affected water content (χ^2^ = 102.9, *p* < 0.0001) but humidity did not (χ^2^ = 1.23, *p* = 0.54). For interaction effects: water content declined faster in 20% RH compared to 80% RH (χ^2^ = 50.0, *p* < 0.025); no significant difference in water loss rate between 40% and 80% RH (χ^2^ = 0.625, *p* = 0.43). All point estimates produced represent the mean from five independent biological samples comprised of 20 pooled females. **b** Survivorship of *yw* adult females aged under RH of 20%, 40%, and 80% (25 °C). Log-rank test of mortality: 20% versus 40%, *p* = 0.0003; 40% versus 80%, *p* = 0.616. Three cages of 125 flies used for each RH condition. **c–e** Relative *PGRP-LC*, *EcR*, and *Defensin* mRNA levels in MTs from *yw* females, mean and SEM, aged at 20%, 40%, and 80% RH (25 °C), sampled at 1, 2, 4, and 6 weeks; three samples of 15 females were censored from demography cages to produce three independent biological samples at each time point and condition. Analysis was performed with Generalized Linear Model Fit by maximum likelihood ratio test for main (age, RH) and interaction (age x RH) effects. *PGRP-LC*: age, χ^2^ = 24.3, *p* < 0.001; RH, χ^2^ = 13.0, *p* = 0.0015; age x RH, χ^2^ = 7.08, *p* = 0.029. *EcR*: age, χ^2^ = 13.3, *p* < 0.001; RH, χ^2^ = 11.3, *p* = 0.0035; age x RH, χ^2^ = 6.75, *p* = 0.034. *Defensin*: age, χ^2^ = 15.1, *p* < 0.001; RH, χ^2^ = 2.79, *p* = 0.095; age x RH, χ^2^ = 4.32, *p* = 0.038

Aged females may produce ecdysone because they are intrinsically water stressed. To explore this hypothesis, cohorts of *yw* adults were maintained in normal food conditions in three levels of relative humidity (RH), namely 20% RH, 40% RH (incubator standard condition), and 80% RH (Additional file 6: Figure S6). Water content was measured in flies sampled from each RH cohort across 7 weeks of age (Fig. 5a). Consistent with reports where water loss rates were directly measured in aging *Drosophila* [16], water content per fly declined with age in all cohorts (χ^2^ = 102.9, *p* < 0.0001), and at a significantly greater rate at 20% RH compared to 80% RH (χ^2^_(age x humidity)_ = 50.02, *p* < 0.025). Lifespan was similar among the 40% and 80% RH cohorts (median life span: 44 and 46 days respectively), but significantly less at 20% RH (median life span: 40 days) (Fig. 5b). Remarkably, elevated RH sharply blunted the increased expression of *PGRP-LC*, *EcR*, and the AMP gene *Defensin* typically observed in the MT of aged adults (Fig. 5c–e). MTs from females at 20% RH strongly upregulated *PGRP-LC*, *EcR*, and *Defensin* by age 6 weeks (age as main effect, each mRNA as independent variable: χ^2^ > 13.0, *p* < 0.001). In contrast, mRNA for *PGRP-LC*, *EcR*, and *Defensin* in cohorts at 80% RH increased to an extent that was significantly less than observed at 20% RH (two-way ANOVA for age and RH, each mRNA: χ^2^_(age x humidity)_ > 4.3, *p* < 0.04). Thus, innate immune aging is blunted in flies reared at RH that reduces intrinsic water stress, decreasing recognition protein expression and AMP gene levels. This amelioration in water loss and immunosenescence also had a modest, but significant, effect on lifespan.

### Desiccation-triggered ecdysone-mediated recognition-protein priming leads to enhanced host defense

**Fig. 6:**
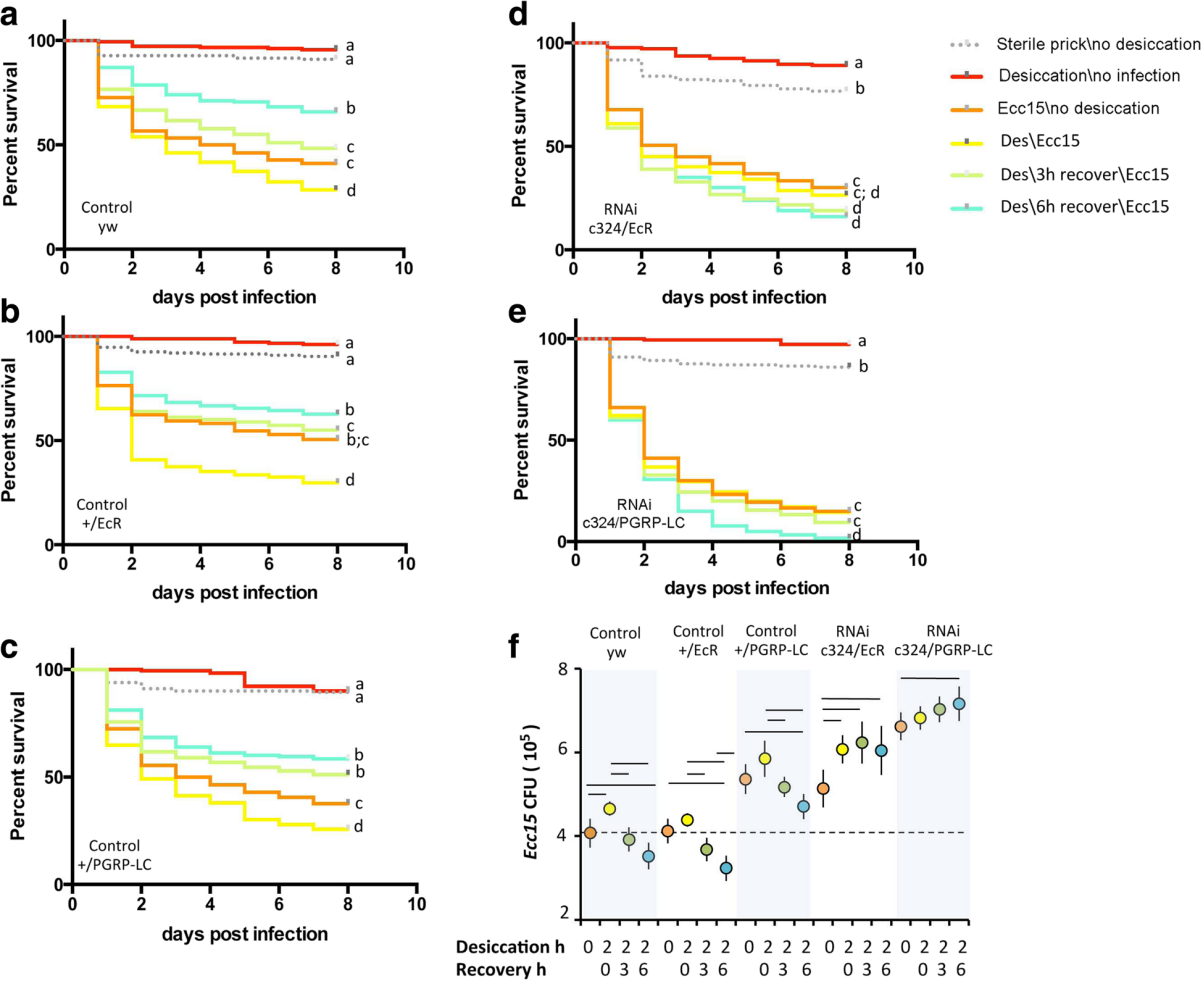
Functional immunity enhanced by desiccation stress response requires *EcR* and *PGRP-LC* in Malpighian tubules (MTs). **a–e** Kaplan*–*Meier survival plots of adult females sterile pricked or infected with *Ecc15*, with or without prior desiccation (des) and either no recovery, 3 h recovery, or 6 h recovery. Genotypes: **a** yw; **b** +/EcR(RNAi); **c** +/PGRP-LC(RNAi); **d** c324-gal4/UAS-EcR(RNAi); **e** c324-gal4/UAS-PGRP-LC(RNAi). Each survival curve is from a combined lifetable of two cohorts tested at different times, each cohort initiated with 90 females. For survival analyses (right censored log-rank test) conducted with six pair-wise comparisons within each genotype; curves with different letters differ significantly by at least *p* < 0.02 (details in Additional file 9: Table S1). **f** Microbial load (colony-forming units) 48 h after *Ecc15* infection of adult females across treatments by desiccation (0 or 2 h) and post-desiccation recovery (0, 3, 6 h). Genotypes as in (**a–e**): wildtype controls (yw, +/EcR, +/PGRP-LC) and *EcR* or *PGRP-LC* knock-down in principal cells (c324 > RNAi). For each genotype, three independent samples were prepared from each treatment group, in both cohort trials (displaying mean and SD of all six observations). CFU compared by ANOVA with Sidak post-hoc comparison, bars indicate differences with *p* < 0.05, see details in Additional file 10: Table S2

We were also interested to examine how recognition-protein priming might alter host defense. To test if desiccation-induced recognition-protein priming affects survival following bacterial infection, we exposed two wild-type fly strains (*wDah* and *yw*) to 2 h desiccation and then immediately infected the adults with the Gram-negative pathogen *Erwinia carotovora carotovora 15* (*Ecc15*). Desiccated flies were significantly more susceptible to *Ecc15* infection compared to those without prior desiccation (Additional file 7: Figure S7). Given that *PGRP-LC* expression is elevated upon desiccation, these results were unexpected and led us to hypothesize that some time, following desiccation, may be required for *PGRP-LC* mRNA to produce functional receptors. Accordingly, we allowed the desiccated flies to recover upon regular food for 3 or 6 h prior to *Ecc15* challenge. Wild-type flies that were allowed to recover for 6 h significantly improved their survival relative to all other infected cohorts, including non-desiccated controls (Fig. 6a–c and Additional file 7: Figure S7). Importantly, this improved survival following septic infection required ecdysone signaling and *PGRP-LC* expression in the MT; knockdown of *EcR* or of *PGRP-LC* in MT principal cells eliminated the survival benefit of desiccation with recovery following *Ecc15* challenge (Fig. 6d, e). These results demonstrate that MTs contribute to host defense, and that recognition-protein priming, specifically in this renal organ, improves outcomes following septic bacterial challenge.

Improved survival upon septic bacterial challenge can arise from tolerance to the infection as well as from immune defenses that reduce microbial load, often referred to as resistance [37]. To determine whether recognition-protein priming affects tolerance or resistance, we measured *Ecc15* colony-forming units (CFUs) from flies treated as in the infection survival studies. Flies were initially inoculated with ~6.0 × 10^5^ CFUs of *Ecc15* (0 h, Additional file 8: Figure S8). At 48 h post infection, wild-type flies harbored less *Ecc15* if they had been desiccated and allowed to recover prior to infection (Fig. 6f), consistent with their improved survival. In contrast, flies with *EcR* or *PGRP-LC* expression knock-down specifically in their MT had a higher *Ecc15* load at 48 h post infection, and desiccation with recovery did not reduce infection load. Thus, recognition-protein priming in adult MTs provides functional whole animal immune resistance to septic bacterial challenge, leading to improved survival.

## Discussion

Water-loss dehydration triggers recognition-protein priming within adult *Drosophila* MTs. This response is mediated by the local synthesis and signaling of ecdysone within MTs, which induces expression of the microbial recognition protein *PGRP-LC*. Elevated *PGRP-LC* potentiates the expression of AMP mRNA in tubules. Increased AMP gene expression is expected to produce more mature AMPs, although the study of the peptides is hampered by a lack of effective antibodies. Our analysis of survival after bacterial challenge with desiccation and recovery suggests that recognition-protein priming in the MT provides functional antimicrobial resistance to the entire animal. Moreover, the load of infecting microbe is reduced in wild-type flies able to mount recognition-protein priming, but not in flies where *PGRP-LC* or *EcR* expression is reduced specifically in MTs, highlighting a previously unknown role for this organ in whole animal antimicrobial defense.

Recognition-protein priming induced by water-loss dehydration may be an adaptive response if dehydration coincides with infection. Observations on this topic are scarce. Virulence in the waxmoth *Galleria mellonella* was increased by infection with *Beauveria bassiana* that exogenously expressed diuretic hormone, which stimulates host water loss [38]. In *Drosophila*, septic infection might increase if water-loss dehydration reduces barrier protection of gut or cuticle [39, 40]. Alternatively, Imd signaling primed by water-loss dehydration could provide novel immune protective or tolerance functions. For instance, Relish (NF-κB) regulates expression of cathepsin-L required for remodeling fat body in the moth *Helicoverpa armigera*, and *Har*-Relish is modulated by ecdysone signaling in this moth [41].

In *Drosophila,* recognition-protein priming is mediated by ecdysone produced within the MT. Water-loss dehydration induces MT to express ecdysone biosynthetic genes and targeted reduction of *dib* within MT stellate or principal cells prevented induction of *PGRP-LC* during water-loss dehydration. How MTs regulate *dib* and other Halloween gene expression in response to water loss is unknown. Desiccation can reduce *Drosophila* neuropeptide capa-1 (homolog of human Neuromedin U) and increase short neuropeptide F and tachykinin in protocerebral neurosecretory cells [42–44]. Capa-1 controls intracellular Ca^2+^ and nitric oxide/guanosine 3′,5′-cyclic monophosphate signaling in MTs [42]. Further, tachykinin induces insulin production and insulin signaling within MTs [45]. Notably, insulin signaling has the capacity to stimulate ecdysone production in the prothoracic gland of developing larvae [46–48]. It remains to be determined whether similar neuroendocrine factors control Halloween gene expression and recognition-protein priming in MTs.

Female *Drosophila* use cholesterol to produce ecdysone in egg follicles. In the fat body, this ecdysone is converted by 20-hydroxylase CYP314A1 (*shade*) to 20E, which activates nuclear hormone receptor EcR and stimulates yolk production [49, 50]. In the ovary, ecdysone also regulates germline stem cells, somatic cysts, and border cells [33]. Other adult tissues in female insects do not produce ecdysone de novo, although some may complete its final synthesis, including the step involving *dib* [51]. Ecdysone synthesis also occurs in adult males, where its production may take place in accessory glands [52–54]. We find that muscle, gut, and MT, express *dib* when adults are dehydrated; these tissues may thus produce ecdysone during physiological stress, or at least complete its synthesis. Consistent with this idea, Rauschenbach et al. [10] and Terashima et al. [11] noted that nutrient restriction and heat increased 20E levels in adult *Drosophila*.

Ecdysone has many potential somatic targets within adult *Drosophila* [24, 30]. Notably, 20E can modulate fly aging. Males and females heterozygous for mutant *EcR* are long-lived, and resistant to the oxidizing agent paraquat as well as to heat and starvation despite being surprisingly fertile [31]. Tricoire et al. [55] subsequently found that mild repression of *EcR* also extended male lifespan, while *EcR* inactivation decreased female lifespan except when egg production was genetically suppressed. Besides control of egg production, ecdysone in adults has stress- and aging-related functions, although where and how these work remain poorly understood.

Here, we studied *Drosophila* innate immune aging in the context of desiccation stress. Water loss may be a typical feature of fly aging. Aged *Drosophila* experience elevated water loss and are less resistant to desiccation stress [56, 57], susceptibilities that may be caused by accelerated cuticle water transpiration [16]. Cuticle lipids regulate barrier transpiration in adult insects and this layer may deteriorate or change composition during aging [15, 58, 59]. We propose that recognition-protein priming and increased AMP response in the aged arise in part because old flies experience high water loss. Consistent with this idea, cohorts maintained at high RH repressed the typical age-related increase in *PGRP-LC*, *Defensin*, and *EcR* expression. Conversely, flies at low RH were somewhat shorter lived, and previous work suggests that chronic innate immune activation reduces life expectancy in an NF-κB-dependent manner [60].

The production of AMPs is costly, impacting physiology and fecundity [61], and is tightly regulated. AMP induction is robust but transient to minimize the deleterious effects of immune activation. Likewise, the Imd pathway, and AMP production, is regulated by ecdysone in the context of development and, as shown here, by stress. This steroid-mediated regulation likely optimizes the AMP response such that the benefits of this mechanism of host defense at least equal these costs. Steroid modulation of AMP induction during development may coordinate this feature of innate immunity with nutrient demands and availability, which change during different growth phases. Alternatively, developmental steroid regulation of AMP production may be an adaptation to the selective pressures associated with the different microbial pathogens encountered with different developmental stages. Furthermore, here we demonstrate that the adult flies use ecdysone to prime innate immune induction of AMPs in the renal organ in response to water-stress imbalance. Remarkably, the resulting elevated expression of AMPs from these renal tissues appears to protect adult animals from systemic, septic infection.

## Conclusions

In this study, we examined how water-loss dehydration increases innate immune responsiveness in *Drosophila* MTs, and how this response improves outcomes following infection and contributes to elevated innate immune gene expression with age. In this model, the stress of water-loss dehydration increased systemic levels of 20E, which is responsible for the pattern recognition-protein priming observed in MTs. While it is not possible to measure 20E levels in a tissue-specific manner, genetic manipulations of both the 20E synthesis gene *dib* and the *EcR*, demonstrate that the tubules are autonomously responding to water-loss dehydration through the production of ecdysone and subsequent sensing of this hormone signal. Interestingly, the data presented here further argue that MTs serve a critical function in anti-bacterial immunity, wherein recognition-protein priming in MTs is required for increased host defense observed after desiccation and recovery, and loss of recognition-protein expression specifically in MTs caused a marked sensitivity to infection. How MTs contribute to immunity and host defense will be the focus of future studies.

In addition, aged flies also display symptoms consistent with water-loss dehydration. Aged flies, reared at 20%, 40%, or 80% RH, were dehydrated, consistent with earlier reports [58]. Aged flies also displayed increased systemic 20E levels and increased expression of the innate immune recognition protein PGRP-LC and effectors. However, adults aged at high relative humidity show less water loss, and have reduced expression of *PGRP-LC*, AMPs, and *EcR* in their tubules. Our study suggests that the steroid hormone ecdysone in old flies is induced in response to age-related water imbalance, and this facilitates immunosenescence in *Drosophila* renal MTs.

## Methods

### *Drosophila* strains

Stocks were reared and maintained at 25 °C, 40% RH and 12-h light/dark cycles. Food media contained cornmeal (5.2%), sugar (11.0%), autolyzed yeast (2.5%; SAF brand) and agar (0.79%) (*w*/*v* in 100 mL water) with 0.2% Tegosept (methyl 4-hydroxybenzoate, Sigma, St. Louis, MO, USA) as an antifungal agent. MT-specific Gal4-driver stocks c724 (stellate cell) and c324 (principal cell) were provided by Julian Dow (University of Glasgow); second chromosomes containing these transgenes were crossed and balanced into a genetic background marked with *yw*, producing *yw*; *c724-Gal4/Cyo* and *yw*; and *c324-Gal4/Cyo*. RNAi to reduce *PGRP-LC*, *EcR*, *Eip75B*, and *dib* was respectively provided from Vienna Stock Center as strains P{UAS-PRGP-LC^KK101636^ RNAi}VIE-260B, P{UAS-EcR^GD1428^RNAi}v37058, P{UAS-Eip75B^KK108982^ RNAi}VIE-260B, and P{UAS-dib^KK106954^RNAi}VIE-260B. Genotypes (F1 offspring) were generated from crosses of these UAS-RNAi stocks with MT Gal4-drivers or by crosses with *yw*. *wDah* was provided by the laboratory of Linda Partridge (University College London).

### 20E treatment

20E was fed to *wDah* females aged 7–10 days old by transferring adults from standard laboratory diet to 1 g fly instant food media with 2 mL of water (Genesee Scientific, San Diego, CA, USA) for 24 h containing vehicle control (20 μL ethanol) or vehicle with 20 μL 1 mM 20E (Sigma cat H-5142).

### Desiccation protocol

Prior to conducting a trial, adult flies were placed in vials with standard food at a density of approximately 15 per vial without anesthesia. Vials were maintained overnight at 25 °C. To initiate each trial, flies from vials were flipped without anesthesia into fresh vials that contained standard food (food control), tissue soaked with 0.5 mL 0.5× PBS solution (PBS control), or nothing (desiccation). MTs were dissected after 2 h of treatment from flies within 5 minutes, immediately transferred to Trizol, and processed for RT-qPCR. Three replicate biological samples of 15 MT (sampled from across five vials) were generated per treatment.

### MT stimulation with TCT

Females (*wDah*) aged 7 to 10 days post eclosion, were processed through the desiccation protocol (with PBS as the only control). Dissected MTs were transferred for 4 h to S2 cell culture medium with TCT (0.01 mM dissolved in water) or without TCT. Expression of *defensin* and *Cec-A2* was measured by qRT-PCR from RNA extracted from these tubules immediately upon dissection (0 h), and at 4 h with or without TCT. Three replicate biological samples were produced for each measure set.

### NanoString nCounter expression analysis

The mRNA content of dissected MTs was analyzed by NanoString methodology according to published procedures [62]. Total RNA (100 ng per sample) was hybridized to the target-specific codeset overnight at 65 °C. The codeset used in Additional file 1: Figure S1 contained probes against a panel of 45 genes encoding AMPs, Imd pathway components, and four loading controls (*nucleostemin*, *alpha tubulin*, *Rp49*, and *Faf2*), while that in Additional file 2: Figure S2 utilized an expanded codeset of 113 immunity-related genes and three loading controls (*nucleostemin*, *alpha tubulin*, and *Faf2*). The hybridized reactions were loaded onto the NanoString Prep station, which removes excess reporter, binds the reporter to the cartridge surface, and stretches the probes for scanning. Subsequently, the cartridges were loaded onto the NanoString Digital Analyzer and scanned. The nCounter data were normalized in two steps. In the first, data was normalized to positive spiked-in controls, as per the manufacturer’s instructions (NanoString Technologies). In the second, the housekeeping genes were used to normalize overall mRNA levels. For Fig. 1a, AMP gene expression was further normalized amongst the four biological replicates by setting the highest value for each gene to 100% and plotted with standard error of the mean. For heat maps in Additional file 1: Figure S1 and Additional file 2: Figure S2, only genes with expression greater than 50 normalized counts in one or more conditions are shown, as lower counts are below the reliable detection threshold for the nCounter.

### Quantitative RT–PCR

Total RNA was extracted from dissected tissue (MT, head, muscle, gut, fat body, ovary) in Trizol reagent (Invitrogen, Carlsbad, CA, USA) and treated with DNase (Ambion). DNase-treated total RNA was quantified with a NanoDrop ND-1000. cDNA was synthesized using iScript cDNA Synthesis (Bio-Rad) and measured on an ABI prism 7300 Sequence Detection System (Applied Biosystems, Carlsbad, CA, USA). For tissue-specific analysis of AMP, *PGRP-LC*, *EcR*, or Halloween genes, three independent biological replicates for each tissue and each treatment group were analyzed. Each replicate included RNA extracted from 10 flies for head, muscle, gut, fat body, and ovary, or 15–20 for MT. Three technical replicates were used for each biological replicate. mRNA abundance of each gene was normalized relative to ribosomal protein L32 (*rp49*) by comparative CT.

### Enzyme immunoassay (EIA) of 20E

Female flies (25–30 flies/sample) were homogenized in 250 μL of ice-cold 100% methanol and centrifuged for 15 min at 18,000 *g* at 4 °C. Supernatants were transferred to 6 × 50 mm borosilicate glass tubes; precipitates were suspended in 200 μL of aqueous 75% methanol and kept on ice for 30 min (adapted from [63]). Samples were centrifuged and dried with a SpeedVac centrifuge. The precipitates were dissolved in EIA buffer at 4 °C and each individual sample was analyzed in a technical triplicate by competitive EIA using 20E EIA antiserum (Cayman Chemical: 482202) and 20E AChE Tracer (Cayman Chemical: 482200). Calibration curves were generated from commercial 20E (Sigma, H5142).

### RH cohorts

Newly enclosed females (*yw*) were mated for 2 days and separated into replicate 1 L demography cages at 125 flies per cage in an environmentally controlled room set at 40% RH, 25 °C, and 12 h light: 12 h dark cycles. Three independent cages (plus supplemental cages for dissection samples) were placed in 60 L clear plastic, tight-lidded storage boxes that maintained RH at 20%, 40%, and 80%. To produce 20% RH, boxes contained a solution of saturated potassium acetate (Fisher Science Cat # P171–500) and solid desiccant (Drierite, W.A. Hammond Co.). To produce 40% RH, the box was left ajar, permitting cages to equilibrate at 40% RH as maintained in the environmental room. To produce 80% RH, boxes contained a solution of saturated ammonium sulfate (Sigma-Aldrich Cat # A4418-500G). Boxes were managed every second day at room temperature and room RH, at which time dead flies were removed and counted. Saturated solutions (and Drierite) were replenished weekly. HOBO data loggers (Onset Computer Corp) continuously monitored RH within boxes; after maintenance, all boxes rapidly re-equilibrated to RH targets (Additional file 5: Figure S5).

Longevity analysis was conducted with JMP statistical software (SAS Institute, Cary, NC, USA). Data from three replicate cages were combined to produce genotype and treatment cohort life tables. Mortality distributions were compared by Log-Rank and proportional hazard analyses. Flies to measure mRNA were maintained in parallel demography cages and sampled at 1, 2, 4, and 6 weeks. MTs were dissected directly from non-anesthetized adults and subjected to RT-qPCR (15 females per biological replicate, three replicates per time point per humidity condition).

Water content was determined from samples in a replicate trial with cohorts at 20%, 40%, and 80% RH. Females were sampled at 1, 2, 4, 6, and 7 weeks from each RH cohort. Point estimates for water content were each generated as the mean among five groups of 20 females; these females were sampled from 8 demography cages, each with 125 females for every RH condition. For each group, females were weighed (Mettler Toledo semi-micron balance), dried overnight at 65 °C, and reweighed. Age, RH treatment, and main-effect interactions were analyzed by generalized linear model likelihood ratio test.

### Infection challenge survival

Adult survival to a controlled infection challenge after desiccation treatment was determined using 100 to 120 flies per group (Additional file 5: Figure S5) or 180 flies per group (Fig. 6) (among two replicate vials). Females at 3 to 7 days old were exposed to food or PBS-only as control, or to 2 h desiccation. Following these treatments, either immediately or after 3 and 6 h recovery on standard food, flies were pricked with a clean microsurgery needle (control) or with a needle dipped into a concentrated pellet of (*Ecc15*) [64, 65]. Surviving flies were subsequently transferred daily to fresh vials and recorded as alive or dead for 8 days. Survival is represented as Kaplan–Meier plots with all pairwise differences evaluated by log-rank test with significance adjusted for multiple comparisons (*p* < 0.01).

### CFU counts

Bacterial load was determined in flies at time 0 and 48 h after infection with *Ecc15*. Individual flies were homogenized in 200 μL of PBS. Homogenates were diluted in series and plated on LB-Ampicillin plates, incubated overnight at 37 °C, and scored for CFU count.

## Additional files

Additional file 1: **Figure S1.** Heat map of differentially expressed innate immune transcripts in Malpighian tubules (MTs). Nanostring nCounter analysis of AMP transcripts from isolated MTs of *wDah* females (7 days old) exposed to TCT for 4 h compared to unstimulated tubules. The mean of four independent biological replicates, harvested on separate days, is shown by heatmap. Scale as indicated in side bar. (PDF 797 kb)

Additional file 2: **Figure S2.** Heat map summarizing innate immune gene expression profiles in desiccated Malpighian tubules (MTs). MTs of 7-day-old *wDah* flies exposed to 2 h desiccation or PBS control treatment were excised, and then either RNA was immediately isolated, or were treated for 4 h with TCT or mock treated, and then RNA was isolated. These RNA samples were used to measure immune-related gene expression by NanoString nCounter. Values shown by heat map represent the mean of four independent experiments. Scale as indicated in side bar. (PDF 847 kb)

Additional file 3: **Figure S3.** Desiccation stress upregulated and amplified TCT-induced expression of 13 AMP genes in Malpighian tubules. Analysis of individual *AMP* gene expression (as indicated), data from nCounter experiments displayed by heat map in Additional file 2: Figure S2. Values represent the mean of four biologically independent replicates, and error bars are standard error of the mean. Statistical analysis was performed by two-way ANOVA and Sidak’s test for pair-wise comparison of desiccation to PBS treatment (*p < 0.05, **p < 0.01, ***p < 0.001, ****p < 0.0001). Side table displays statistical metrics after Bonferroni correction for multiple comparisons for the interaction of desiccation and TCT treatments from the same ANOVA analyses. (PDF 631 kb)

Additional file 4: **Figure S4.** mRNA expression of Halloween genes. Halloween genes (*spook*, *phantom*, *shadow*, and *shade*) were measured in adult tissues from young (7 days) and old (40 days) females. All values normalized relative to head samples from young adults in food control group. The mean of three independent biological replicates is shown. (PDF 2424 kb)

Additional file 5: **Figure S5.** RNAi on *disembodied* (*dib*) in Malpighian tubules (MTs). Reduced *dib* mRNA in MTs when *dib* RNAi is driven in stellate (*c724 > dib* RNAi) or principal (*c324 > dib* RNAi) cells relative to control (*ywT1/dib* RNAi). Results shown represent the mean and SEM of three independent replicates. (PDF 897 kb)

Additional file 6: **Figure S6.** Relative humidity (RH) and temperature recorded from demography chambers under different humidity conditions. Spikes indicate when chambers were opened to access cages and show rapid homeostasis of the humidity control system. Blue tracings show realized RH, black tracing represents temperature. (PDF 3450 kb)

Additional file 7: **Figure S7.** Impact of desiccation followed by recovery on the survival of wild-type flies infected with *Erwinia carotovora carotovora 15* (*Ecc15*). Kaplan–Meier survival of *yw* and *wDah* adult females challenged with *Ecc15*. Uninfected flies were desiccated or not for 2 h and survival was monitored for 8 days. Other cohorts were challenged with *Ecc15* infection with or without a prior 2 h desiccation treatment and recovery of 0, 3, or 6 h prior, as indicated. Plots represent the survival kinetics of 100–120 files, combined from two separate trials, for each genotype; cohorts with significantly different mortality are grouped with different letters (all cases, log-rank test *p* < 0.05, see side tables). (PDF 1741 kb)

Additional file 8: **Figure S8.** Initial bacterial loads in *Erwinia carotovora carotovora 15* (*Ecc15*)-infected flies. Adult load (colony-forming units) of *Ecc15* at 0 h post infection as a function of desiccation (0 or 2 h) and post-desiccation recovery (0, 3, 6 h); among three control genotypes (*yw*, *+/EcR*, *+/PGRP-LC*) and genotypes where *EcR* or *PGRP-LC* were knocked down by RNAi in principal cells (c324 > RNAi), no significant differences were observed at 0 h. Results represent the mean of six assays and the error bars show standard deviation. (PDF 1024 kb)

Additional file 9: **Table S1.** Statistical analysis for the effect of desiccation on survival to *Ecc15* infection with or without recovery treatment, supporting Fig. 6a-e. (DOCX 26 kb)

Additional file 10: **Table S2.** Statistical analysis for the effect of desiccation on bacterial growth after *Erwinia carotovora carotovora 15* infection with or without recovery treatment, supporting Fig. 6f. (DOCX 15 kb)

## Abbreviations

20E: 20-hydroxyecdysone
AMP: antimicrobial peptide
*Cec-A2*: Cecropin-A2
CFU: colony-forming unit
DAP-type PGN: diaminopimelic acid type-peptidoglycan
*dib*: *disembodied*
*Ecc15*: *Erwinia carotovora carotovora 15*
EcR: Ecdysone receptor
*Eip75*: *ecdysone induced protein 75*
MTs: Malpighian tubules
NF-κB: nuclear factor kappa-light-chain-enhancer of activated B cells
PGRP: Peptidoglycan recognition protein
RH: relative humidity
TCT: tracheal cytotoxin

## References

[1] Lemaitre B, Hoffmann J (2007). The host defense of Drosophila melanogaster. Annu Rev Immunol.

[2] Buchon N, Silverman N, Cherry S (2014). Immunity in Drosophila melanogaster--from microbial recognition to whole-organism physiology. Nat Rev Immunol.

[3] Kaneko T, Yano T, Aggarwal K, Lim JH, Ueda K, Oshima Y, Peach C, Erturk-Hasdemir D, Goldman WE, Oh BH (2006). PGRP-LC and PGRP-LE have essential yet distinct functions in the drosophila immune response to monomeric DAP-type peptidoglycan. Nat Immunol.

[4] Kleino A, Ramia NF, Bozkurt G, Shen Y, Nailwal H, Huang J, Napetschnig J, Gangloff M, Chan FK, Wu H (2017). Peptidoglycan-sensing receptors trigger the formation of functional amyloids of the adaptor protein IMD to initiate Drosophila NF-kappaB signaling. Immunity.

[5] Paquette N, Broemer M, Aggarwal K, Chen L, Husson M, Erturk-Hasdemir D, Reichhart JM, Meier P, Silverman N (2010). Caspase-mediated cleavage, IAP binding, and ubiquitination: linking three mechanisms crucial for Drosophila NF-kappaB signaling. Mol Cell.

[6] Chen L, Paquette N, Mamoor S, Rus F, Nandy A, Leszyk J, Shaffer SA, Silverman N (2017). Innate immune signaling in Drosophila is regulated by transforming growth factor beta (TGFbeta)-activated kinase (Tak1)-triggered ubiquitin editing. J Biol Chem.

[7] Stoven S, Silverman N, Junell A, Hedengren-Olcott M, Erturk D, Engstrom Y, Maniatis T, Hultmark D (2003). Caspase-mediated processing of the Drosophila NF-kappaB factor Relish. Proc Natl Acad Sci U S A.

[8] Rus F, Flatt T, Tong M, Aggarwal K, Okuda K, Kleino A, Yates E, Tatar M, Silverman N (2013). Ecdysone triggered PGRP-LC expression controls Drosophila innate immunity. EMBO J.

[9] Meister M, Richards G (1996). Ecdysone and insect immunity: the maturation of the inducibility of the diptericine gene in Drosophila larvae. Insect Biochemistry Mol Biol.

[10] Rauschenbach IY, Sukhanova MZ, Hirashima A, Sutsugu E, Kuano E (2000). Role of the ecdysteroid system in the regulation of Drosophila reproduction under environmental stress. Dokl Biol Sci.

[11] Terashima J, Takaki K, Sakurai S, Bownes M (2005). Nutritional status affects 20-hydroxyecdysone concentration and progression of oogenesis in Drosophila melanogaster. J Endocrinol.

[12] Ishimoto H, Kitamoto T (2010). The steroid molting hormone Ecdysone regulates sleep in adult Drosophila melanogaster. Genetics.

[13] Ishimoto H, Kitamoto T (2011). Beyond molting--roles of the steroid molting hormone ecdysone in regulation of memory and sleep in adult Drosophila. Fly.

[14] Dow JA (2009). Insights into the Malpighian tubule from functional genomics. J Exp Biol.

[15] Nghiem D, Gibbs AG, Rose MR, Bradley TJ (2000). Postponed aging and desiccation resistance in Drosophila melanogaster. Exp Gerontol.

[16] Gibbs AG, Markow TA (2001). Effects of age on water balance in Drosophila species. Physiol Biochem Zool.

[17] Tzou P, Ohresser S, Ferrandon D, Capovilla M, Reichhart JM, Lemaitre B, Hoffmann JA, Imler JL (2000). Tissue-specific inducible expression of antimicrobial peptide genes in Drosophila surface epithelia. Immunity.

[18] McGettigan J, McLennan RK, Broderick KE, Kean L, Allan AK, Cabrero P, Regulski MR, Pollock VP, Gould GW, Davies SA (2005). Insect renal tubules constitute a cell-autonomous immune system that protects the organism against bacterial infection. Insect Biochem Mol Biol.

[19] Kaneko T, Goldman WE, Mellroth P, Steiner H, Fukase K, Kusumoto S, Harley W, Fox A, Golenbock D, Silverman N (2004). Monomeric and polymeric gram-negative peptidoglycan but not purified LPS stimulate the Drosophila IMD pathway. Immunity.

[20] Hoffmann JA (2003). The immune response of Drosophila. Nature.

[21] Werner T, Liu G, Kang D, Ekengren S, Steiner H, Hultmark D (2000). A family of peptidoglycan recognition proteins in the fruit fly Drosophila melanogaster. Proc Natl Acad Sci U S A.

[22] Chang CI, Ihara K, Chelliah Y, Mengin-Lecreulx D, Wakatsuki S, Deisenhofer J (2005). Structure of the ectodomain of Drosophila peptidoglycan-recognition protein LCa suggests a molecular mechanism for pattern recognition. Proc Natl Acad Sci U S A.

[23] Chang CI, Pili-Floury S, Herve M, Parquet C, Chelliah Y, Lemaitre B, Mengin-Lecreulx D, Deisenhofer J (2004). A Drosophila pattern recognition receptor contains a peptidoglycan docking groove and unusual L,D-carboxypeptidase activity. PLoS Biol.

[24] Schwedes CC, Carney GE (2012). Ecdysone signaling in adult Drosophila melanogaster. J Insect Physiol.

[25] Dimarcq J-L, Imler J-L, Lanot R, Ezekowitz RAB, Hoffmann JA, Janeway CA, Lagueux M (1997). Treatment of l(2)mbn Drosophila tumorous blood cells with the steroid hormone ecdysone amplifies the inducibility of antimicrobial peptide gene expression. Insect Biochem Mol Biol.

[26] Beckstead RB, Lam G, Thummel CS (2005). The genomic response to 20-hydroxyecdysone at the onset of Drosophila metamorphosis. Genome Biol.

[27] Silverman N, Zhou R, Stoven S, Pandey N, Hultmark D, Maniatis T (2000). A Drosophila IkappaB kinase complex required for Relish cleavage and antibacterial immunity. Genes Dev.

[28] Flatt T, Heyland A, Rus F, Porpiglia E, Sherlock C, Yamamoto R, Garbuzov A, Palli SR, Tatar M, Silverman N (2008). Hormonal regulation of the humoral innate immune response in Drosophila melanogaster. J Exp Biol.

[29] Koelle MR, Talbot WS, Segraves WA, Bender MT, Cherbas P, Hogness DS (1991). The Drosophila EcR gene encodes an ecdysone receptor, a new member of the steroid receptor superfamily. Cell.

[30] Schwedes C, Tulsiani S, Carney GE (2011). Ecdysone receptor expression and activity in adult Drosophila melanogaster. J Insect Physiol.

[31] Simon AF, Shih C, Mack A, Benzer S (2003). Steroid control of longevity in *Drosophila melanogaster*. Science.

[32] Ishimoto H, Wang Z, Rao Y, Wu CF, Kitamoto T (2013). A novel role for ecdysone in Drosophila conditioned behavior: linking GPCR-mediated non-canonical steroid action to cAMP signaling in the adult brain. PLoS Genet.

[33] Belles X, Piulachs MD (2015). Ecdysone signalling and ovarian development in insects: from stem cells to ovarian follicle formation. Biochim Biophys Acta.

[34] Pletcher S, Macdononald SJ, Marguerie R, Certa U, Stearns SC, Goldstein DB, Partridge L (2002). Genome_wide transcript profiles in aging and calorically restricted Drosophila melanogaster. Curr Biol.

[35] Zerofsky M, Harel E, Silverman N, Tatar M (2005). Aging of the innate immune response in Drosophila melanogaster. Aging Cell.

[36] Landis GN, Abdueva D, Skvortsov D, Yang J, Rabin BE, Carrick J, Tavare S, Tower J (2004). Similar gene expression patterns characterize aging and oxidative stress in Drosophila melanogaster. Proc Natl Acad Sci U S A.

[37] Schneider DS, Ayres JS (2008). Two ways to survive infection: what resistance and tolerance can teach us about treating infectious diseases. Nat Rev Immunol.

[38] Fan Y, Borovsky D, Hawkings C, Ortiz-Urquiza A, Keyhani NO (2012). Exploiting host molecules to augment mycoinsecticide. Virulence.

[39] Rera M, Clark RI, Walker DW (2012). Intestinal barrier dysfunction links metabolic and inflammatory markers of aging to death in Drosophila. Proc Natl Acad Sci U S A.

[40] Guo L, Karpac J, Tran SL, Jasper H (2014). PGRP-SC2 promotes gut immune homeostasis to limit commensal dysbiosis and extend lifespan. Cell.

[41] Zhang Y, Lu YX, Liu J, Yang C, Feng QL, Xu WH (2013). A regulatory pathway, ecdysone-transcription factor relish-cathepsin L, is involved in insect fat body dissociation. PLoS Genet.

[42] Terhzaz S, Cabrero P, Robben JH, Radford JC, Hudson BD, Milligan G, Dow JA, Davies SA (2012). Mechanism and function of Drosophila capa GPCR: a desiccation stress-responsive receptor with functional homology to human neuromedinU receptor. PLoS One.

[43] Kean L, Cazenave W, Costes L, Broderick KE, Graham S, Pollock VP, Davies SA, Veenstra JA, Dow JA (2002). Two nitridergic peptides are encoded by the gene capability in Drosophila melanogaster. Am J Physiol Regul Integr Comp Physiol.

[44] Kahsai L, Kapan N, Dircksen H, Winther AM, Nassel DR (2010). Metabolic stress responses in Drosophila are modulated by brain neurosecretory cells that produce multiple neuropeptides. PLoS One.

[45] Soderberg JA, Birse RT, Nassel DR (2011). Insulin production and signaling in renal tubules of Drosophila is under control of tachykinin-related peptide and regulates stress resistance. PLoS One.

[46] Smith WA, Lamattina A, Collins M (2014). Insulin signaling pathways in lepidopteran ecdysone secretion. Front Physiol.

[47] Colombani J, Bianchini L, Layalle S, Pondeville E, Dauphin-Villemant C, Antoniewski C, Carre C, Noselli S, Leopold P (2005). Antagonistic actions of ecdysone and insulins determine final size in Drosophila. Science.

[48] Mirth C (2005). Ecdysteroid control of metamorphosis in the differentiating adult leg structures of Drosophila melanogaster. Dev Biol.

[49] Truman JW, Riddiford LM (2002). Endocrine insights into the evolution of metamorphosis in insects. Annu Rev Entomol.

[50] Buszczak M, Freeman MR, Carlson JR, Bender M, Cooley L, Segraves WA (1999). Ecdysone response genes govern egg chamber development during mid-oogenesis in Drosophila. Development.

[51] Rewitz KF, Rybczynski R, Warren JT, Gilbert LI (2006). Identification, characterization and developmental expression of Halloween genes encoding P450 enzymes mediating ecdysone biosynthesis in the tobacco hornworm, Manduca sexta. Insect Biochem Mol Biol.

[52] Hentze JL, Moeller ME, Jorgensen AF, Bengtsson MS, Bordoy AM, Warren JT, Gilbert LI, Andersen O, Rewitz KF (2013). Accessory gland as a site for prothoracicotropic hormone controlled ecdysone synthesis in adult male insects. PLoS One.

[53] Handler AM (1982). Ecdysteroid titers during pupal and adult development in Drosophila melanogaster. Dev Biol.

[54] Ishimoto H, Sakai T, Kitamoto T (2009). Ecdysone signaling regulates the formation of long-term courtship memory in adult Drosophila melanogaster. Proc Natl Acad Sci U S A.

[55] Tricoire H, Battisti V, Trannoy S, Lasbleiz C, Pret AM, Monnier V (2009). The steroid hormone receptor EcR finely modulates Drosophila lifespan during adulthood in a sex-specific manner. Mech Ageing Dev.

[56] Huthinson EW, Mackinley MD, Rose MR, Service PM (1985). Resistance to environmental stress in Drosophila melanogaster selected for postponed senescence. Physiol Zool.

[57] Drapeau MD, Gass EK, Simison MD, Mueller LD, Rose MR (2000). Testing the heterogeneity theory of late-life mortality plateaus by using cohorts of Drosophila melanogaster. Exp Gerontol.

[58] Gibbs A, Mousseau TA, Crowe JH (1991). Genetic and acclimatory variation in biophysical properties of insect cuticle lipids. Proc Natl Acad Sci USA.

[59] Kuo TH, Yew JY, Fedina TY, Dreisewerd K, Dierick HA, Pletcher SD (2012). Aging modulates cuticular hydrocarbons and sexual attractiveness in Drosophila melanogaster. J Exp Biol.

[60] Libert S, Chao Y, Chu X, Pletcher SD (2006). Trade-offs between longevity and pathogen resistance in Drosophila melanogaster are mediated by NFkappaB signaling. Aging Cell.

[61] Schwenke RA, Lazzaro BP, Wolfner MF (2016). Reproduction-immunity trade-offs in insects. Annu Rev Entomol.

[62] Geiss GK, Bumgarner RE, Birditt B, Dahl T, Dowidar N, Dunaway DL, Fell HP, Ferree S, George RD, Grogan T (2008). Direct multiplexed measurement of gene expression with color-coded probe pairs. Nat Biotechnol.

[63] Hackney JF, Zolali-Meybodi O, Cherbas P (2012). Tissue damage disrupts developmental progression and ecdysteroid biosynthesis in Drosophila. PLoS One.

[64] Basset A, Khush RS, Braun A, Gardan L, Boccard F, Hoffmann JA, Lemaitre B (2000). The phytopathogenic bacteria Erwinia carotovora infects Drosophila and activates an immune response. Proc Natl Acad Sci U S A.

[65] Zaidman-Remy A, Herve M, Poidevin M, Pili-Floury S, Kim MS, Blanot D, Oh BH, Ueda R, Mengin-Lecreulx D, Lemaitre B (2006). The Drosophila amidase PGRP-LB modulates the immune response to bacterial infection. Immunity.

