# Supplementary figures and images for: Dehydration triggers ecdysone-mediated recognition-protein priming and elevated anti-bacterial immune responses in Drosophila Malpighian tubule renal cells
Source: BMC Biol. 2018 May 31;16:60. doi: 10.1186/s12915-018-0532-5 (PMC5984326; doi:10.1186/s12915-018-0532-5)

Figure S1

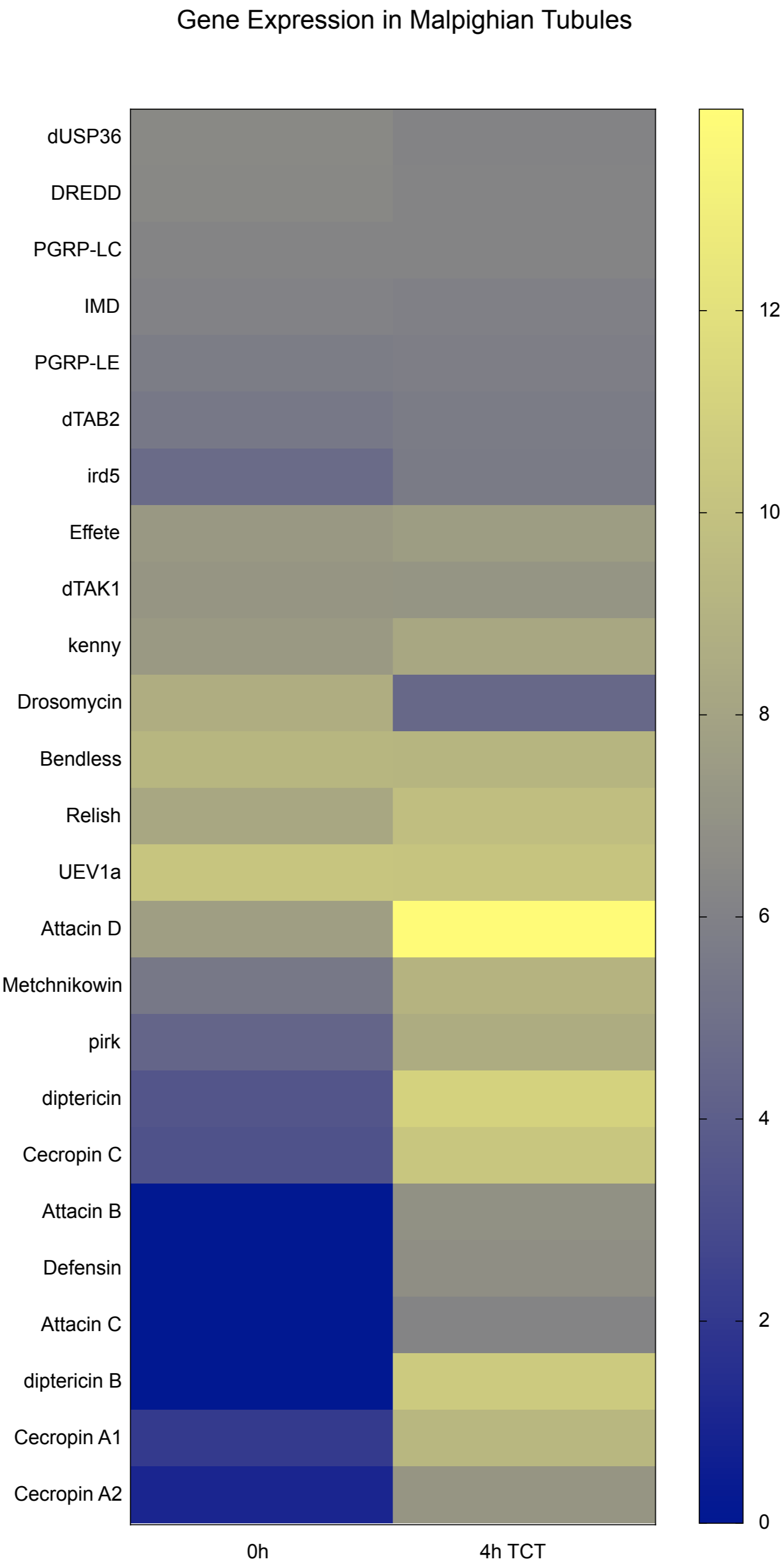

Supplement: Supplementary file 1 — Figure S1. Heat map of differentially expressed innate immune transcripts in Malpighian tubules (MTs). Nanostring nCounter analysis of AMP transcripts from isolated MTs of wDah females (7 days old) exposed to TCT for 4 h compared to unstimulated tubules. The mean of four independent biological replicates, harvested on separate days, is shown by heatmap. Scale as indicated in side bar. (PDF 797 kb) [file 12915_2018_532_MOESM1_ESM.pdf]

Figure S2

Gene Expression in Malpighian Tubules

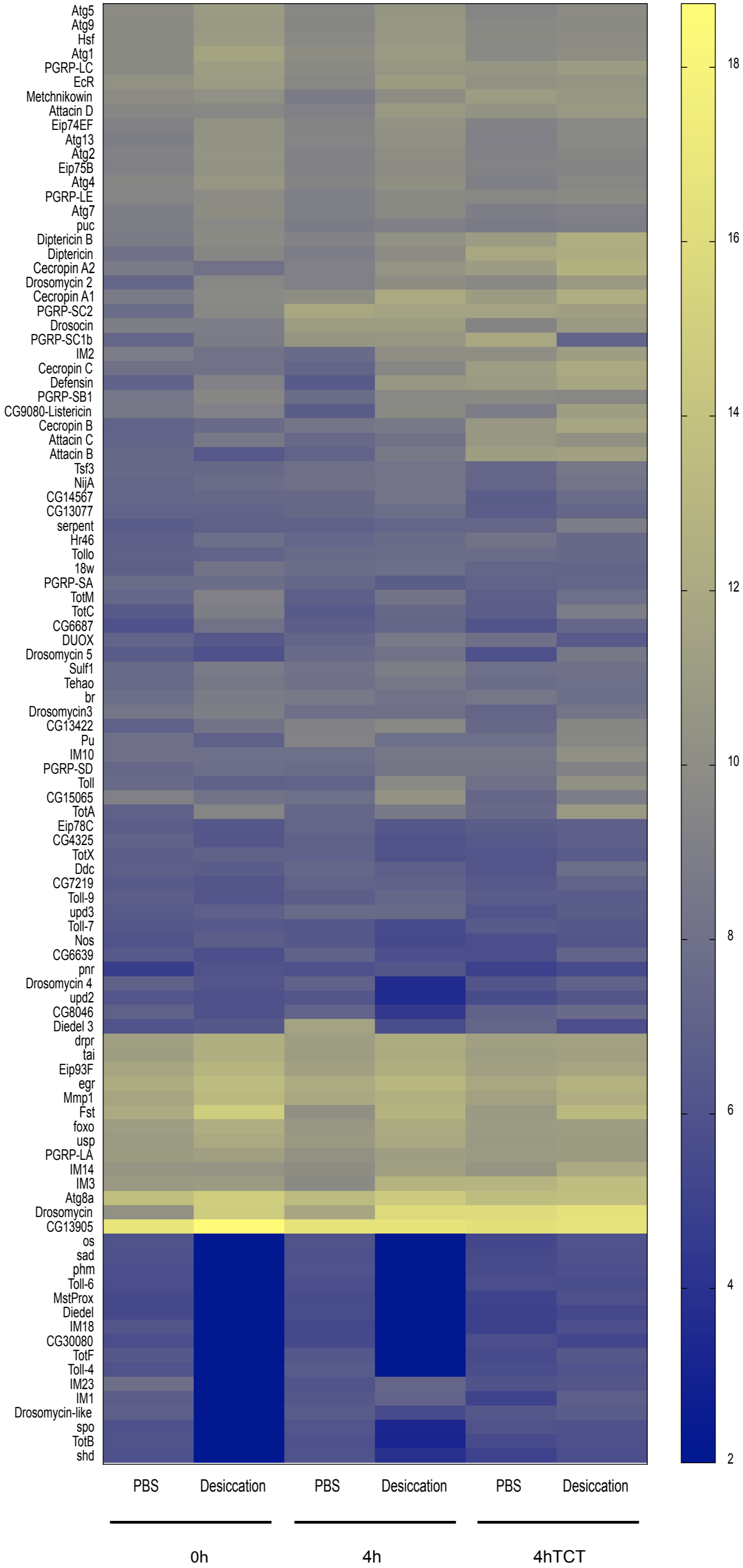

Supplement: Supplementary file 2 — Figure S2. Heat map summarizing innate immune gene expression profiles in desiccated Malpighian tubules (MTs). MTs of 7-day-old wDah flies exposed to 2 h desiccation or PBS control treatment were excised, and then either RNA was immediately isolated, or were treated for 4 h with TCT or mock treated, and then RNA was isolated. These RNA samples were used to measure immune-related gene expression by NanoString nCounter. Values shown by heat map represent the mean of four independent experiments. Scale as indicated in side bar. (PDF 847 kb) [file 12915_2018_532_MOESM2_ESM.pdf]

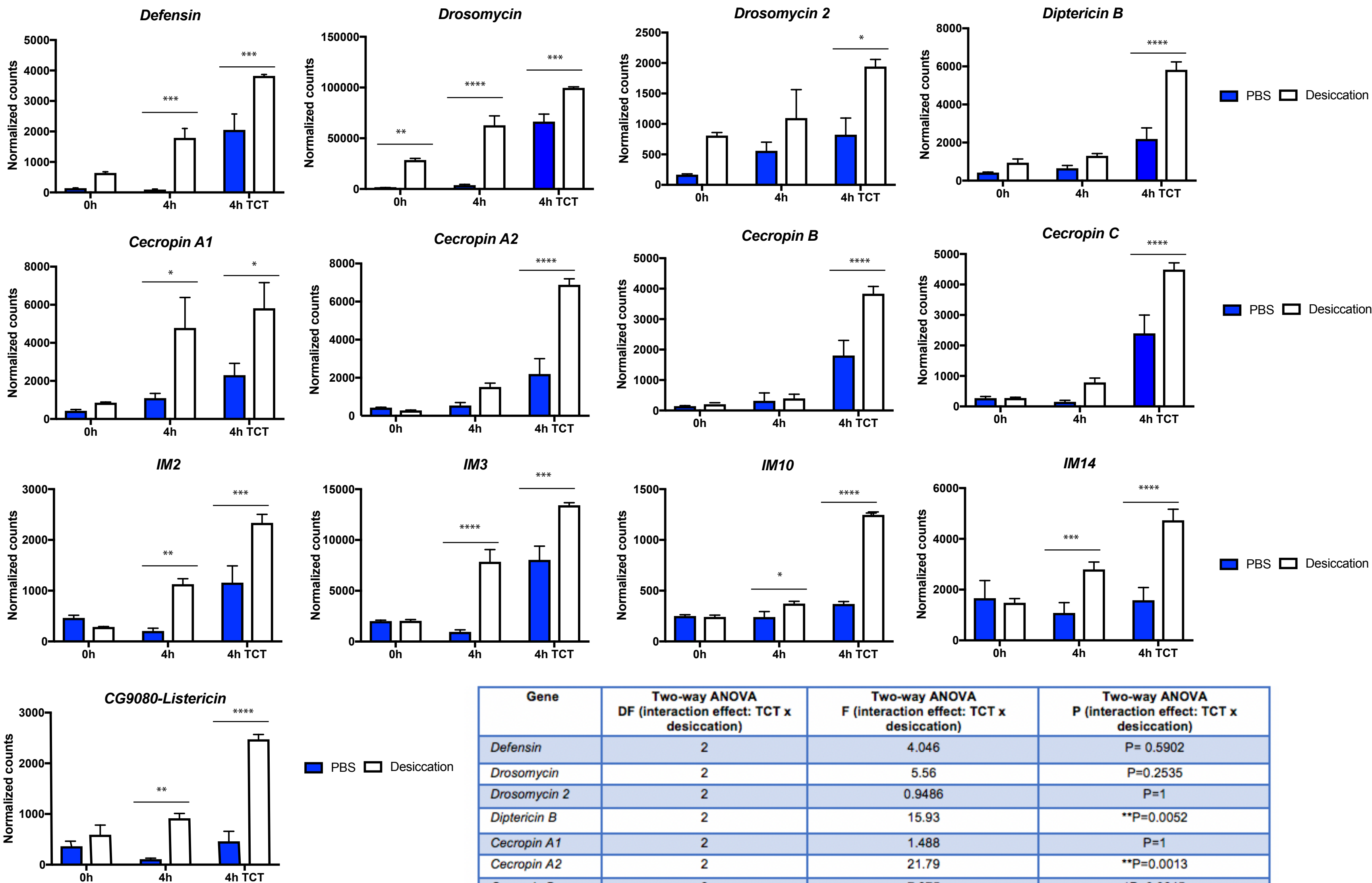

Figure S3

Supplement: Supplementary file 3 — Figure S3. Desiccation stress upregulated and amplified TCT-induced expression of 13 AMP genes in Malpighian tubules. Analysis of individual AMP gene expression (as indicated), data from nCounter experiments displayed by heat map in Additional file 2: Figure S2. Values represent the mean of four biologically independent replicates, and error bars are standard error of the mean. Statistical analysis was performed by two-way ANOVA and Sidak’s test for pair-wise comparison of desiccation to PBS treatment (*p < 0.05, **p < 0.01, ***p < 0.001, ****p < 0.0001). Side table displays statistical metrics after Bonferroni correction for multiple comparisons for the interaction of desiccation and TCT treatments from the same ANOVA analyses. (PDF 631 kb) [file 12915_2018_532_MOESM3_ESM.pdf]

Figure S4

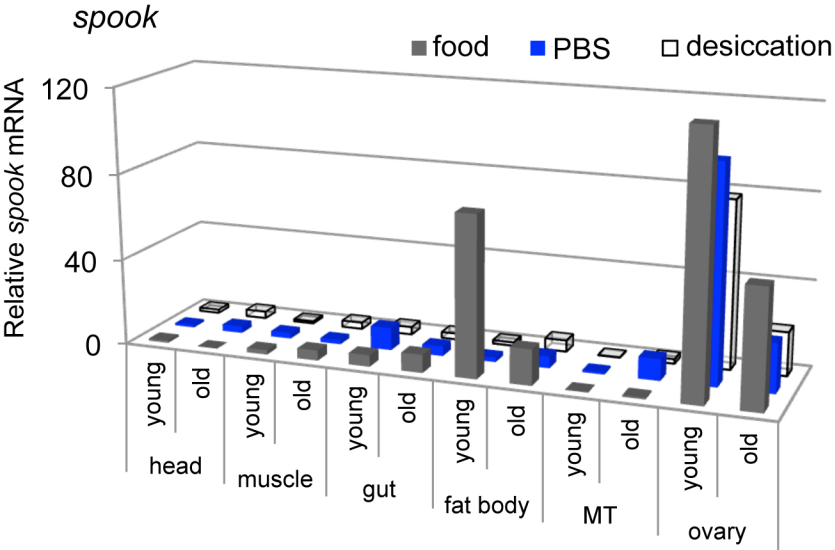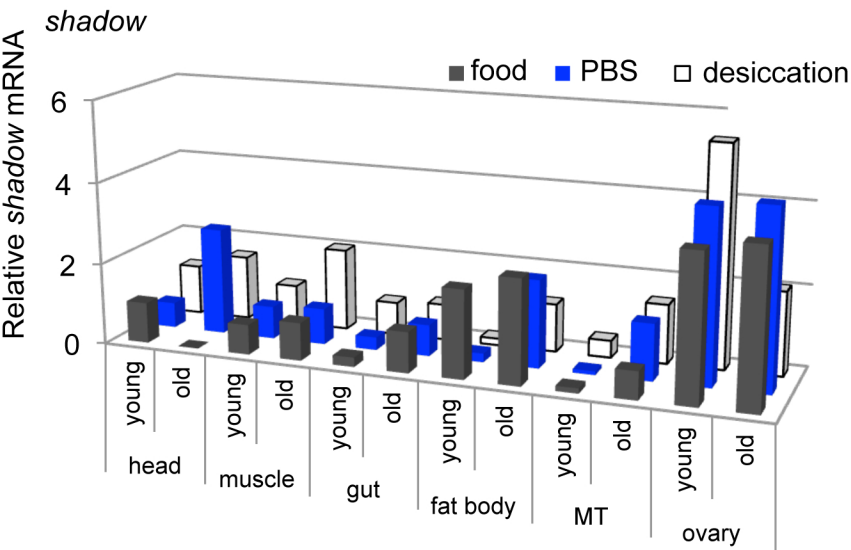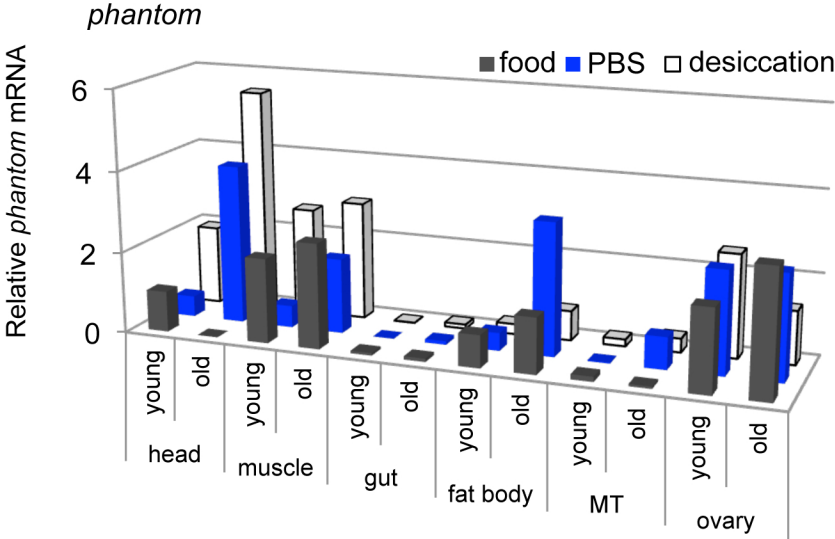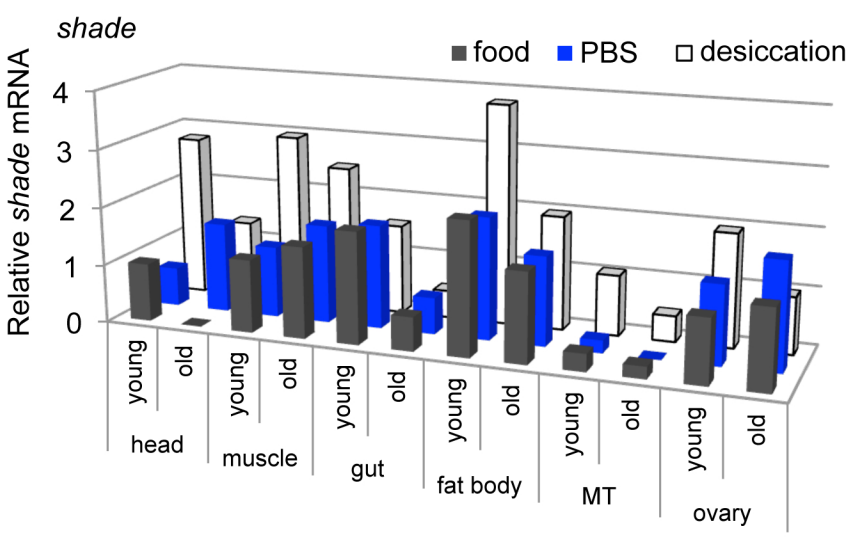

Supplement: Supplementary file 4 — Figure S4. mRNA expression of Halloween genes. Halloween genes (spook, phantom, shadow, and shade) were measured in adult tissues from young (7 days) and old (40 days) females. All values normalized relative to head samples from young adults in food control group. The mean of three independent biological replicates is shown. (PDF 2424 kb) [file 12915_2018_532_MOESM4_ESM.pdf]

**Figure S5**

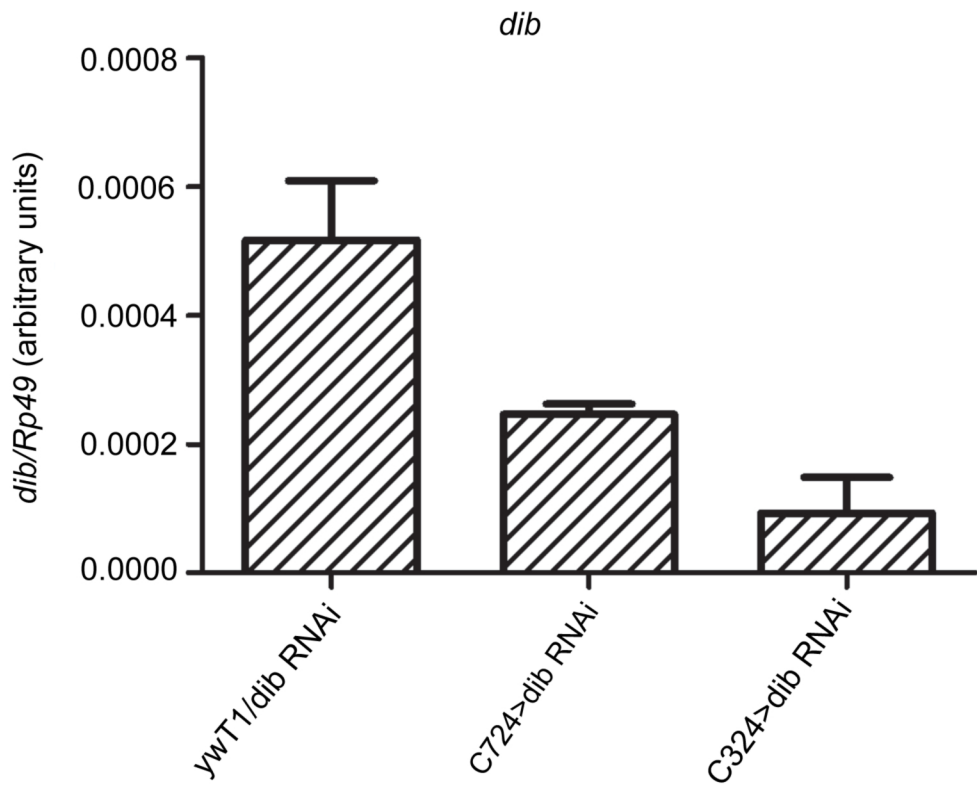

Supplement: Supplementary file 5 — Figure S5. RNAi on disembodied (dib) in Malpighian tubules (MTs). Reduced dib mRNA in MTs when dib RNAi is driven in stellate (c724 > dib RNAi) or principal (c324 > dib RNAi) cells relative to control (ywT1/dib RNAi). Results shown represent the mean and SEM of three independent replicates. (PDF 897 kb) [file 12915_2018_532_MOESM5_ESM.pdf]

Figure S6

20%RH

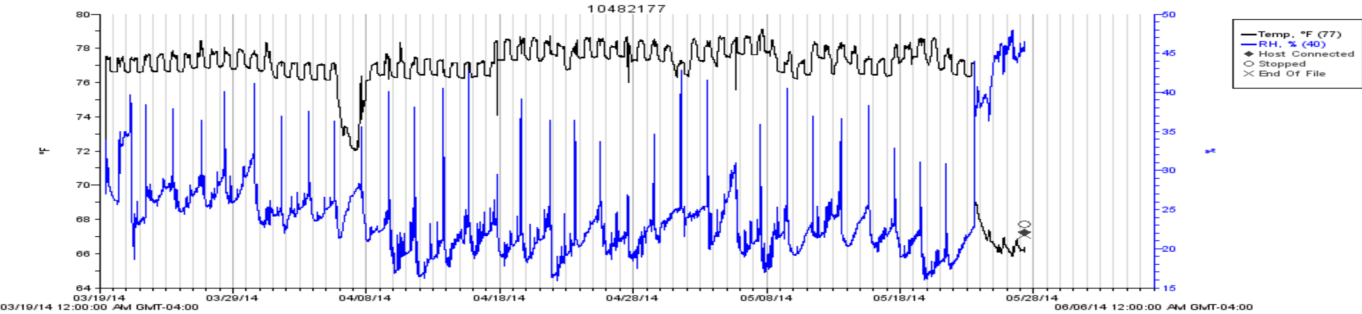

40%RH

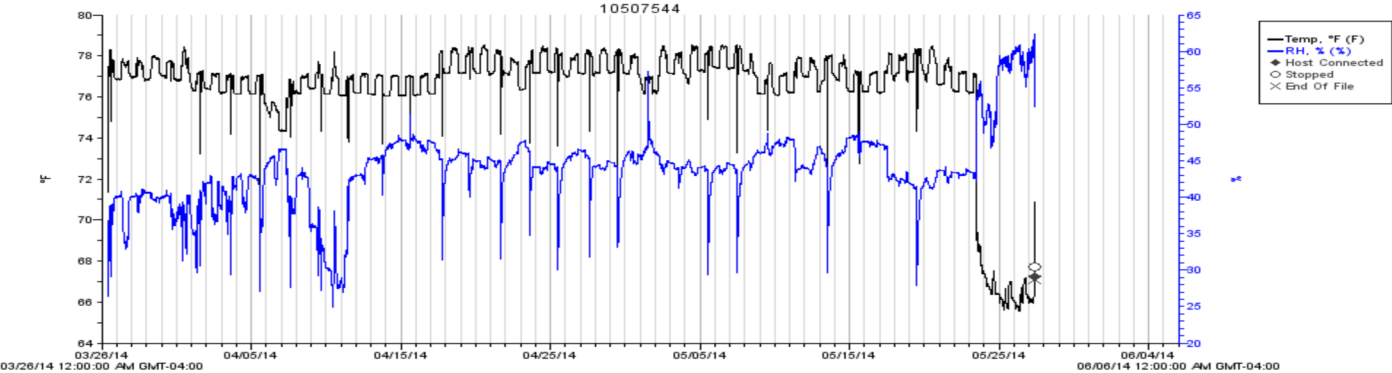

80%RH

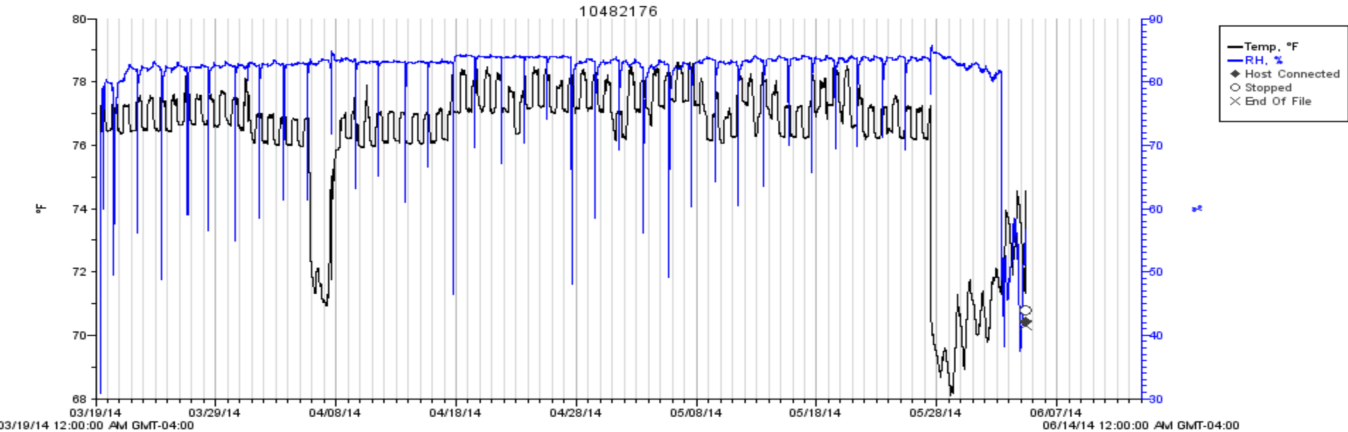

Supplement: Supplementary file 6 — Figure S6. Relative humidity (RH) and temperature recorded from demography chambers under different humidity conditions. Spikes indicate when chambers were opened to access cages and show rapid homeostasis of the humidity control system. Blue tracings show realized RH, black tracing represents temperature. (PDF 3450 kb) [file 12915_2018_532_MOESM6_ESM.pdf]

Figure S8

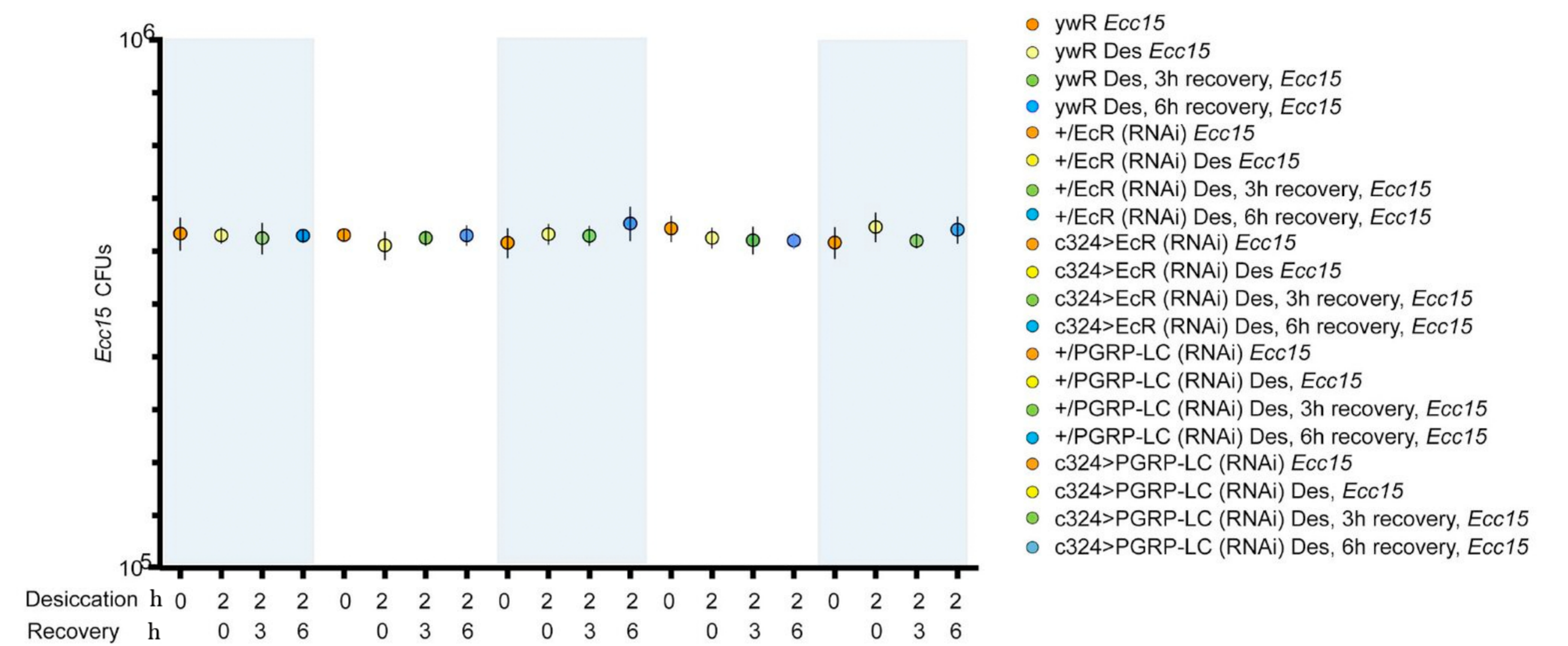

Supplement: Supplementary file 8 — Figure S8. Initial bacterial loads in Erwinia carotovora carotovora 15 (Ecc15)-infected flies. Adult load (colony-forming units) of Ecc15 at 0 h post infection as a function of desiccation (0 or 2 h) and post-desiccation recovery (0, 3, 6 h); among three control genotypes (yw, +/EcR, +/PGRP-LC) and genotypes where EcR or PGRP-LC were knocked down by RNAi in principal cells (c324 > RNAi), no significant differences were observed at 0 h. Results represent the mean of six assays and the error bars show standard deviation. (PDF 1024 kb) [file 12915_2018_532_MOESM8_ESM.pdf]
