# Supplementary material for: Dehydration triggers ecdysone-mediated recognition-protein priming and elevated anti-bacterial immune responses in Drosophila Malpighian tubule renal cells
Source: BMC Biol. 2018 May 31;16:60. doi: 10.1186/s12915-018-0532-5 (PMC5984326; doi:10.1186/s12915-018-0532-5)
Supplement: Supplementary file 7 — Figure S7. Impact of desiccation followed by recovery on the survival of wild-type flies infected with Erwinia carotovora carotovora 15 (Ecc15). Kaplan–Meier survival of yw and wDah adult females challenged with Ecc15. Uninfected flies were desiccated or not for 2 h and survival was monitored for 8 days. Other cohorts were challenged with Ecc15 infection with or without a prior 2 h desiccation treatment and recovery of 0, 3, or 6 h prior, as indicated. Plots represent the survival kinetics of 100–120 files, combined from two separate trials, for each genotype; cohorts with significantly different mortality are grouped with different letters (all cases, log-rank test p < 0.05, see side tables). (PDF 1741 kb) [file 12915_2018_532_MOESM7_ESM.pdf]

Figure S7

A)

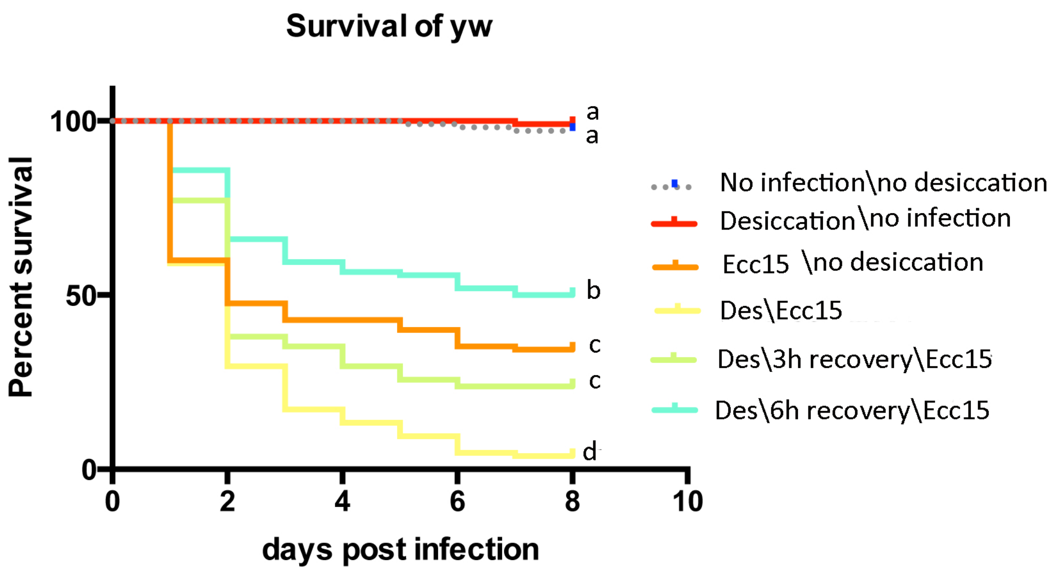

Treatment comparison	p value	Hazard ratio Ratio (and its reciprocal)
Ecc15 vs Des +*Ecc15*	p<0.0001	0.3939; 2.539
Des+*Ecc15* vs Des +*Ecc15* 3h recovery	p<0.0001	2.156; 0.4639
Des+*Ecc15* vs Des +*Ecc15* 6h recovery	p<0.0001	5.002; 0.1999

B)

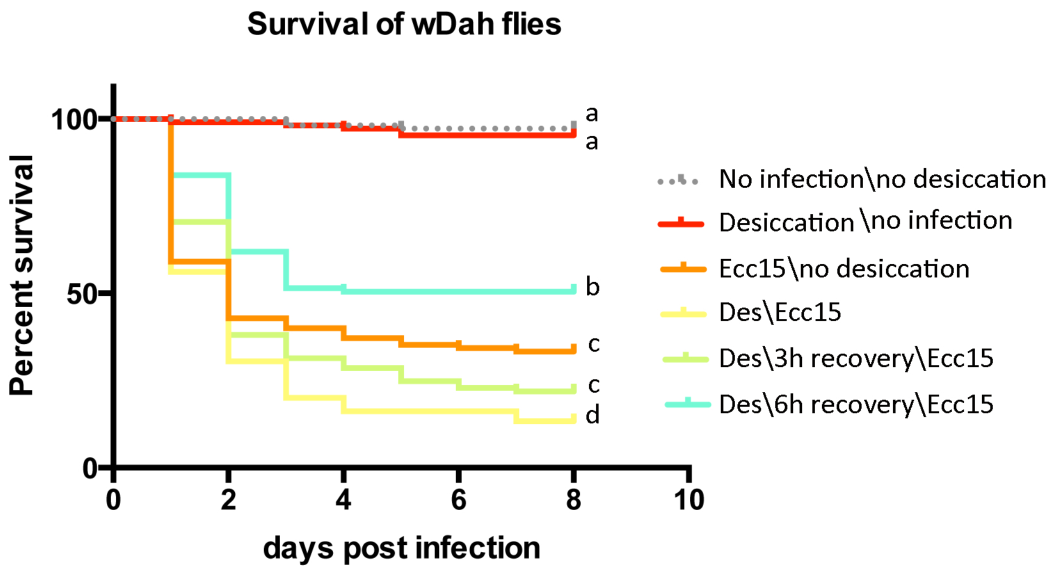

Treatment comparison	p value	Hazard ratio Ratio (and its reciprocal)
Ecc15 vs Des +*Ecc15*	p=0.0037	0.5635; 1.775
Des+*Ecc15* vs Des +*Ecc15* 3h recovery	p=0.039	1.481; 0.6751
Des+*Ecc15* vs Des +*Ecc15* 6h recovery	p<0.0001	3.435; 0.2911
