# Supplementary material for: Dehydration triggers ecdysone-mediated recognition-protein priming and elevated anti-bacterial immune responses in Drosophila Malpighian tubule renal cells
Source: BMC Biol. 2018 May 31;16:60. doi: 10.1186/s12915-018-0532-5 (PMC5984326; doi:10.1186/s12915-018-0532-5)
Supplement: Supplementary file 9 — Table S1. Statistical analysis for the effect of desiccation on survival to Ecc15 infection with or without recovery treatment, supporting Fig. 6a-e. (DOCX 26 kb) [file 12915_2018_532_MOESM9_ESM.docx]

**Table S1**

Statistical analysis for the effect of desiccation on survival to *Ecc15* infection with or without recovery treatment, supporting Figure 6A-E

| Genotype | Treatment comparison | p (Log-rank Mantel-Cox test) | Hazard ratio  (Mantel-Haenszel)  Ratio (and its reciprocal) |
| --- | --- | --- | --- |
| ywR | *Ecc15* vs Des+*Ecc15* | *p=0.02 | 0.7187; 1.391 |
|  | *Ecc15* vs Des+3h recovery+*Ecc15* | ns | 1.262; 0.7924 |
|  | *Ecc15* vs Des+6hr recovery*+Ecc15* | ****p<0.0001 | 2.242; 0.4460 |
|  | Des+*Ecc15* vs Des+3hr recovery *+Ecc15* | ***p=0.0001 | 1.769; 0.5653 |
|  | Des+*Ecc15* vs Des+6hr recovery+*Ecc15* | ****p<0.0001 | 3.117; 0.320 |
|  | Des+3h recovery +*Ecc15* vs Des+6h recovery + *Ecc15* | ***p=0.0008 | 1.786; 0.5600 |
| +/EcR (RNAi) | *Ecc15* vs Des+*Ecc15* | ****p<0.0001 | 0.5233; 1.911 |
|  | *Ecc15* vs Des+3h recovery+*Ecc15* | ns | 1.135; 0.8808 |
|  | *Ecc15* vs Des+6hr recovery*+Ecc15* | *p=0.0225 | 1.496; 0.6683 |
|  | Des+*Ecc15* vs Des+3hr recovery *+Ecc15* | ****p<0.0001 | 2.159; 0.4632 |
|  | Des+*Ecc15* vs Des+6hr recovery+*Ecc15* | ****p<0.0001 | 2.852; 0.3506 |
|  | Des+3h recovery +*Ecc15* vs Des+6h recovery + *Ecc15* | ns | 1.318; 07588 |
| +/PGRP-LC (RNAi) | *Ecc15* vs Des+*Ecc15* | *p=0.01 | 0.7086; 1.411 |
|  | *Ecc15* vs Des+3h recovery+*Ecc15* | *p=0.0197 | 1.452; 0.6885 |
|  | *Ecc15* vs Des+6hr recovery*+Ecc15* | ***p=0.0002 | 1.845; 0.5420 |
|  | Des+*Ecc15* vs Des+3hr recovery *+Ecc15* | ****p<0.0001 | 2.047; 0.4885 |
|  | Des+*Ecc15* vs Des+6hr recovery+*Ecc15* | ****p<0.0001 | 2.581; 0.3875 |
|  | Des+3h recovery +*Ecc15* vs Des+6h recovery + *Ecc15* | ns | 1.272; 0.7862 |
| c324>EcR (RNAi) | *Ecc15* vs Des+*Ecc15* | ns | 0.8676; 1.153 |
|  | *Ecc15* vs Des+3h recovery+*Ecc15* | **p=0.0071 | 1.500; 0.6667 |
|  | *Ecc15* vs Des+6hr recovery*+Ecc15* | **p=0.0031 | 0.6591; 1.517 |
|  | Des+*Ecc15* vs Des+3hr recovery *+Ecc15* | ns | 0.7877; 1.270 |
|  | Des+*Ecc15* vs Des+6hr recovery+*Ecc15* | ns | 0.7602; 1.315 |
|  | Des+3h recovery +*Ecc15* vs Des+6h recovery + *Ecc15* | ns | 0.9788; 1.022 |
| c324>PGRP-LC (RNAi) | *Ecc15* vs Des+*Ecc15* | ns | 0.9513; 1.051 |
|  | *Ecc15* vs Des+3h recovery+*Ecc15* | ns | 0.8024; 1.246 |
|  | *Ecc15* vs Des+6hr recovery*+Ecc15* | ****p<0.0001 | 0.5543; 1.804 |
|  | Des+*Ecc15* vs Des+3hr recovery *+Ecc15* | ns | 0.5868; 1.704 |
|  | Des+*Ecc15* vs Des+6hr recovery+*Ecc15* | ***p=0.002 | 0.8457; 1.183 |
|  | Des+3h recovery +*Ecc15* vs Des+6h recovery + *Ecc15* | **p=0.009 | 0.6903; 1.449 |
