# Supplementary material for: Dehydration triggers ecdysone-mediated recognition-protein priming and elevated anti-bacterial immune responses in Drosophila Malpighian tubule renal cells
Source: BMC Biol. 2018 May 31;16:60. doi: 10.1186/s12915-018-0532-5 (PMC5984326; doi:10.1186/s12915-018-0532-5)
Supplement: Supplementary file 10 — Table S2. Statistical analysis for the effect of desiccation on bacterial growth after Erwinia carotovora carotovora 15 infection with or without recovery treatment, supporting Fig. 6f. (DOCX 15 kb) [file 12915_2018_532_MOESM10_ESM.docx]

**Table S2**

Statistical analysis for the effect of desiccation on bacterial growth after *Ecc15* infection with or without recovery treatment, supporting Figure 6F.

| **Treatment comparison**  **(48h post infection)**  **One-way ANOVA**  **Sidak’s multiple comparison test** | **ywR** | **+/EcR (RNAi)** | **+/PGRP-LC (RNAi)** | **c324>EcR (RNAi)** | **c324>PGRP-LC (RNAi)** |
| --- | --- | --- | --- | --- | --- |
| *Ecc15* vs Des+*Ecc15* | p<0.05 | ns | ns | p<0.0001 | ns |
| *Ecc15* vs Des+3h recovery+*Ecc15* | ns | ns | ns | p<0.0001 | ns |
| *Ecc15* vs Des+6hr recovery*+Ecc15* | p<0.05 | p<0.0001 | p<0.01 | p<0.0001 | p<0.05 |
| Des+*Ecc15* vs Des+3hr recovery *+Ecc15* | p<0.001 | p=0.0001 | p<0.01 | ns | ns |
| Des+*Ecc15* vs Des+6hr recovery+*Ecc15* | p<0.0001 | p<0.0001 | p<0.0001 | ns | ns |
| Des+3h recovery +*Ecc15* vs Des+6h recovery + *Ecc15* | ns | p<0.05 | ns | ns | ns |
